# Enhanced Organic Photocatalysis in Confined Flow through a Carbon Nitride Nanotube Membrane with Conversions in the Millisecond Regime

**DOI:** 10.1021/acsnano.0c09661

**Published:** 2021-04-06

**Authors:** Yajun Zou, Kai Xiao, Qing Qin, Jian-Wen Shi, Tobias Heil, Yevheniia Markushyna, Lei Jiang, Markus Antonietti, Aleksandr Savateev

**Affiliations:** † State Key Laboratory of Electrical Insulation and Power Equipment, Center of Nanomaterials for Renewable Energy, School of Electrical Engineering, Xi’an Jiaotong University, Xi’an 710049, People’s Republic of China; ‡ Department of Colloid Chemistry, Max Planck Institute of Colloids and Interfaces, Am Mühlenberg 1, 14476 Potsdam, Germany; § Key Laboratory of Bio-inspired Materials and Interfacial Science, Technical Institute of Physics and Chemistry, Chinese Academy of Sciences, Beijing 100190, People’s Republic of China

**Keywords:** carbon nitride, nanotube, confined photocatalysis, nanometer flow reactors, enhanced flow

## Abstract

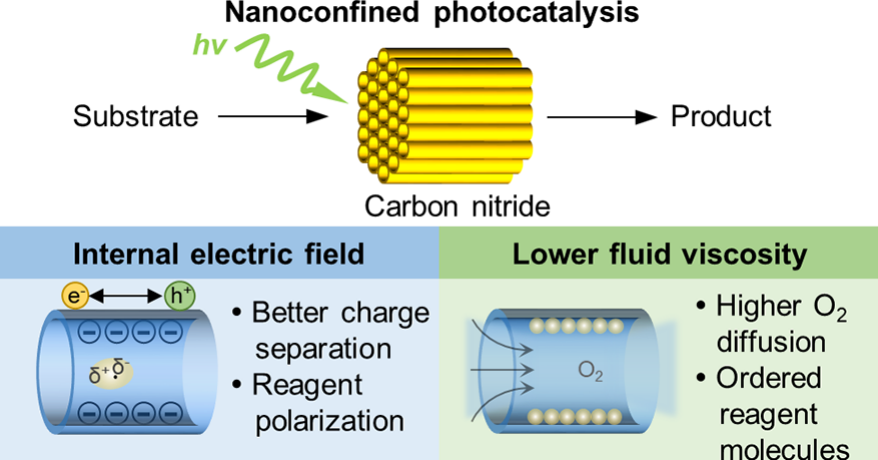

Bioinspired nanoconfined catalysis has developed to become an important tool for improving the performance of a wide range of chemical reactions. However, photocatalysis in a nanoconfined environment remains largely unexplored. Here, we report the application of a free-standing and flow-through carbon nitride nanotube (CNN) membrane with pore diameters of 40 nm for confined photocatalytic reactions where reactants are in contact with the catalyst for <65 ms, as calculated from the flow. Due to the well-defined tubular structure of the membrane, we are able to assess quantitatively the photocatalytic performance in each of the parallelized single carbon nitride nanotubes, which act as spatially isolated nanoreactors. In oxidation of benzylamine, the confined reaction shows an improved performance when compared to the corresponding bulk reaction, reaching a turnover frequency of (9.63 ± 1.87) × 10^5^ s^–1^. Such high rates are otherwise only known for special enzymes and are clearly attributed to the confinement of the studied reactions within the one-dimensional nanochannels of the CNN membrane. Namely, a concave surface maintains the internal electric field induced by the polar surface of the carbon nitride inside the nanotube, which is essential for polarization of reagent molecules and extension of the lifetime of the photogenerated charge carriers. The enhanced flow rate upon confinement provides crucial insight on catalysis in such an environment from a physical chemistry perspective. This confinement strategy is envisioned not only to realize highly efficient reactions but also to gain a fundamental understanding of complex chemical processes.

Nanoconfined catalysis has emerged as a viable strategy for achieving challenging chemical transformations.^1^ It offers means to isolate both catalytic sites and reactant molecules in nanosized cavities, where catalytic reactions behave significantly different from those observed in bulk systems.^2^ Previous studies reveal that a confined environment induces a change in energetics and kinetics of catalytic reactions by imposing specific orientations and conformations on reactant molecules.^3^ Confinement effects give rise to higher activity, improved selectivity, catalyst stabilization, and better catalyst recovery and recyclability.^4^ These advantages are envisioned to be applicable to all types of catalytic transformations, with the final goal of overcoming some of the drawbacks in conventional catalytic systems.

A variety of different types of nanoreactors incorporating catalytic sites have been exploited to perform space-confined reactions, such as nanopores or nanoholes in porous architectures,^5−7^ nanochannels in tubular structures,^8−10^ and van der Waals gaps in layered materials.^11−13^ Among them, special attention has been directed to one-dimensional (1D) nanochannels because they possess appealing properties that make them ideal flow nanoreactors.^14^ A “quantum-confined superfluidity” (QSF) phenomenon was recently reported by Wen *etal*.,^15,16^ where the reactant fluid exhibits an ultrafast mass transport behavior within nanochannels. Liu *etal*. revealed that reactant molecules could pass through nanochannels in certain orientation and molecular configuration.^17^ These phenomena just illustrate the opportunities for nanochannels to act as confinement platforms in important chemical transformations. On the other hand, it has been already shown that catalytic reactions are highly sensitive to the diameter and length of the nanochannels, which offers a way to realize controllable nanoconfined catalysis.^18^

Currently, catalytic transformations using nanochannels are mostly performed in the defined cavities of carbon nanotubes (CNTs), porous metal oxides, and silica molecular sieves as reaction chambers in batch mode.^8,19,20^ For example, Feng *etal*. realized photooxidation of olefins in Pt(II) complex loaded SBA-15 mesoporous molecular sieves.^21^ Chen *etal*. reported enhanced Fischer–Tropsch synthesis with Fe catalysts confined in CNTs.^10^ More recently, Gao *etal*. developed Ni-Al_2_O_3_ nanotubes as confined nanoreactors for the hydrogenation of cinnamaldehyde.^8^ However, from a practical application perspective, the synthetic protocols needed to generate desired nanostructures are still, in most cases, quite costly and low yielding. Despite tremendous advances in semiconductor photocatalysis related to synthesis of value-added organic compounds,^22−27^ a major challenge in this area is to develop catalytic tools that are easily recovered and reused so as to be compatible with setups for larger scale production.

Carbon nitride is an attractive semiconducting material bearing multiple advantages, such as low price and simplicity of preparation of various nanostructures with specific morphologies, together with high stability and reusability, and has shown its efficacy as a photocatalyst for water splitting, carbon dioxide reduction, and many other organic conversions under visible light irradiation.^28−31^ In order to intensify the process and make it compatible with industrial needs, already now several approaches to perform carbon nitride photocatalysis in flow have been proposed, such as using photoreactor tubes packed with carbon nitride powder^32,33^ or carbon nitride coated glass beads,^34^ serial microbatch reactors (SMBRs),^35^ employing nanoparticles of potassium poly(heptazine imide) in quasi-homogeneous fashion,^36^ and oscillatory plug flow photoreactors.^37^ Based on this rich experience base, it looks like an ideal candidate to analyze the specific effects of nanoconfined catalysis, here photocatalysis. Although several reports have described using carbon nitride nanotubes as photocatalysts,^38−42^ these nanotubes are randomly oriented and have not been assembled into free-standing devices to mediate confined photocatalytic reactions in flow.

Recently, a chemical vapor deposition (CVD) approach was put forward, which allows applying partly nanometer thick homogeneous layers conformally on other surfaces.^43^ Such a CVD coating was also applied on porous anodic aluminum oxide (AAO) membranes.^44^ The functionality of the compound device was illustrated by creating a highly performing light-driven ion pump.^45^ In this example, a surface charge gradient induced by incident photons drives ion transport within the asymmetric carbon nitride nanotubes, giving a readable ionic current output.

Herein, we utilize an ensemble of vertically oriented 1D carbon nitride nanochannels fabricated with the aid of a commercial AAO sheet as visible light responsive nanoreactors to perform confined photocatalytic reactions in a flow mode (Scheme 1). The photocatalytic activity of this “nanoreactor bundle” is then quantified in the degradation of methylene blue and oxidation of benzylic amines under visible light irradiation.

**Scheme 1:**
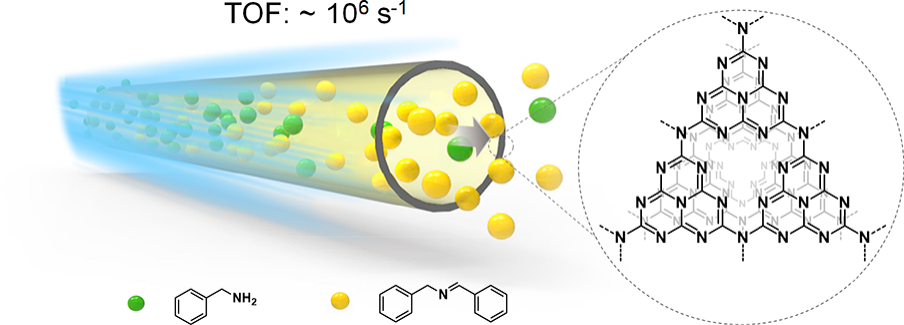
Schematic representation of flow photocatalytic reactions occurring in the carbon nitride channels. The green and yellow spheres represent reactant and product molecules, respectively.

## Discussion

**Figure 1:**
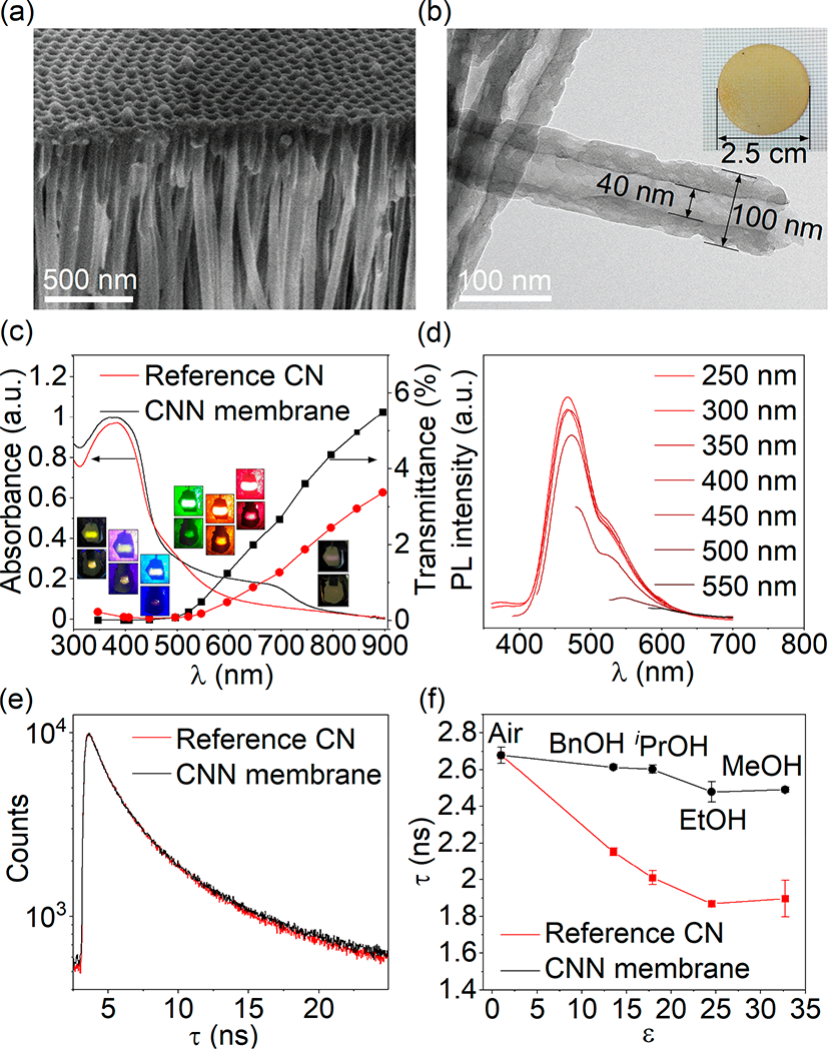
(a) SEM image of the CNN membrane. (b) TEM image of the CNN membrane. Inset is a photo of the CNN membrane. (c) Diffuse reflectance UV–vis absorbance spectra and wavelength-dependent transmittance spectra of the CNN membrane and the reference CN tablet. Inset images show the appearance of the CNN membrane (top) and the reference CN (bottom) upon irradiation with quasi monochromatic light. Light source was located behind the carbon nitride material. (d) PL spectra of the CNN membrane obtained using excitation light of different wavelengths. (e) TRES of the CNN membrane and reference CN acquired under excitation with λ = 375 nm in air. (f) Dependence of carbon nitride average fluorescence lifetime (τ) on relative permittivity of the medium (ε).

The CNN membrane was synthesized based on our previous work with CVD using melamine as a starting material and an AAO membrane as the substrate.^45−47^ A scanning electron microscope (SEM) image shows that carbon nitride is conformally deposited within the nanochannels of the AAO membrane throughout the membrane thickness (Figure S1a,b). A pure CNN membrane can then be obtained by etching the AAO template with acid. It is evident from the SEM image (Figure 1a) that the template was completely removed, producing highly ordered nanotube arrays that constitute the membrane. The photo in the inset in Figure 1b demonstrates that the membrane with a 2.5 cm diameter is self-supporting and therefore can function as a permeable layer in a flow photoreactor. Moreover, the CNN membrane is semitransparent (inset in Figure 1b), which ensures more homogeneous illumination.

Figure 1b shows the transmission electron microscopy (TEM) image of the CNN membrane. The single nanotube displays an external diameter of ∼100 nm, an inner diameter of ∼40 nm, and a wall thickness of ∼30 nm, while the length is defined by the CNN membrane thickness, i.e., 0.2 mm. The X-ray diffraction (XRD) pattern of the CNN membrane is similar to that of carbon nitride powder obtained during the calcination of melamine (Figure S2), with two identical peaks at 13.0° and 27.3° corresponding to (100) interplanar packing of heptazine units and a (002) π–π interlayer stacking motif, respectively.^48^

The optical properties of the CNN membrane were investigated as follows. For comparison, a CN tablet was prepared by pressing bulk carbon nitride powder obtained during CNN membrane fabrication into a tablet of the same thickness as the CNN membrane (∼0.2 mm). In the UV–vis absorbance spectra of these two materials a band at wavelength (λ) < 460 nm due to π–π* transitions was observed (Figure 1c). Notably, the CNN membrane exhibited an additional broad absorption band in the visible light region with a tail extending up to 800 nm. The optical band gap (BG) of the CNN membrane was determined from a Tauc plot (Figure S3), taking the π–π* transitions as the principal band for calculation. The calculated BG value is 2.63 eV, which is consistent with the reported results of graphitic carbon nitride (g-C_3_N_4_).^49,50^

For quantifying the optical efficiencies of the setup, the transmittance (*T*) of the CNN membrane and the reference CN tablet was calculated by eq 1:
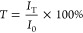
1where *T* is transmittance, *I*_0_ is light intensity before passing through the sample, mW cm^–2^; and *I*_T_ is light intensity after passing through the sample, mW cm^–2^. Unlike diffuse reflectance measurements, the data shown in Figure 1b (connected data points) also take into account light scattering. Thus, absorption at λ > 800 nm is mainly due to scattering, while the absorption coefficient (*A*) at 800 nm was calculated to be 26 ± 0.005 and 24 ± 0.004 mW cm^–3^ for the CNN membrane and the reference CN tablet, respectively, using eq 2 (see the *A* at other λ in Table S1):
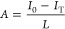
2where *A* is the absorption coefficient, mW cm^–3^; *I*_0_ is light intensity before passing through the sample, mW cm^–2^; *I*_T_ is light intensity after passing through the sample, mW cm^–2^; and *L* is the thickness of the CNN membrane, cm.

The CNN membrane demonstrates slightly higher light transmittance than the CN tablet in the range 525–900 nm that is due to the presence of regularly packed nanotubes. This is also supported by the naked-eye observation of the CNN membrane and the reference CN tablet upon irradiation with quasi monochromatic light (insets in Figure 1c), where a much larger and brighter light spot and thereby less diffuse scattering were observed for the CNN membrane. The semitransparency allows for more homogeneous penetration of incident light into the membrane.

The steady-state photoluminescence (PL) spectra of the CNN membrane were measured using different excitation λ ranging from 250 to 550 nm (Figure 1d). A strong emission peak centered at 468 nm due to the band-to-band recombination of electrons and holes was observed. Another minor emission peak centered at approximately 520 nm indicates a recombination through luminescence centers with lower energies, which speak for some intraband states that could come from the inevitable intrinsic defects in carbon nitride generated in the thermal CVD process.^51,52^ With the increase of excitation wavelength λ, the emission peak position shows a slight red-shift. The emission hardly occurs upon excitation above 500 nm because the λ goes out of the main absorption band (Figure 1c).

The interface between the solid and electrolyte plays a crucial role in semiconductor photocatalysis. Thus, band bending in the space charge region facilitates separation of the photogenerated charges.^53^ On the other hand, the ionic strength of the electrolyte and relative permittivity (ε) of the medium define charge density and potential at the surface of the semiconductor,^54^ which in turn may also be viewed as a type of internal electric field that facilitates separation of the charges (Supplementary Discussion 1).^55^ We investigated changes in the excited-state dynamics of carbon nitride induced by the confinement effect using time-resolved emission spectroscopy (TRES) in media with ε ranging from 1 (air) to 111 (formamide) (Figure S4). In air, the average fluorescence lifetimes (τ) of the CNN membrane and reference CN are the same, 2.68 ns. When immersed into a liquid medium, the corresponding τ of the CNN membrane is higher compared to the reference carbon nitride regardless of the nature of the medium, which (in combination with a fluorescence quantum efficiency of *ca*. 3%) implies higher probability of the redox reaction to take place. Upon an increase of ε, τ shows nonmonotonic behavior, which we explain by the interference of the redox properties of the media. Given that the valence band (VB) potential of carbon nitride is +1.95 V *vs* reversible hydrogen electrode (RHE), some of the used liquids can serve as electron donors and as a result reduce the lifetime of the photogenerated carriers. To take this feature into account, herein we limit our discussion to benzyl alcohol, ^*i*^PrOH, EtOH, and MeOH, which have similar oxidation potentials.^36^ Thus, the τ of the reference CN and the CNN membrane gradually decreases with the increasing permittivity of the medium (Figure 1f). The trend supports the hypothesis that higher permittivity of the medium induces dissociation of polar surface groups and increases the density of charges on the surface, which in turn enhances the magnitude of the internal electric field and therefore facilitates separation of charge carriers in the space charge region. Holes, as positively charged species, migrate to negatively charged domains on the surface of carbon nitride.

To reveal the electronic band structure of the CNN membrane, Mott–Schottky analysis was performed. As shown in Figure S5, the Mott–Schottky plot has a positive slope, indicating n-type semiconductor behavior. This enables us to take the flat band potential (*E*_fb_) approximately as the conduction band (CB) position. The CB position of the CNN membrane is determined to be −1.36 V *versus* Ag/AgCl, corresponding to −0.75 V *versus* RHE. The VB position calculated by the addition of the band gap energy to the CB value is +1.95 V *versus* RHE.

In classical heterogeneous photocatalytic reactions with a suspended catalyst, the conversion is usually limited by poor light penetration due to the strong absorption by the particles. In this photoreactor setup, the structure is more homogeneous, which allows shorter effective light paths and thereby deeper light penetration. Nevertheless, the transmittance is only a few percent, which makes the illuminated side of the membrane more active. All the following discussions thereby have to be taken with this gradient-of-light argument.

Another point to discuss is that catalysis in confined environments can suffer from product inhibition, if the product displays high affinity to the catalyst.^56^ In our case, 1D carbon nitride nanochannels support ion and electron transport very well and prevent further pressure buildup throughout the reactions, as nicely shown in the optical ion-pumping experiments.^47^ Another advantage of membrane flow reactors as such is that the reaction mixture is immediately separated from the catalyst without the necessity to perform filtration or centrifugation after well-defined contact times; that is, it is one of the most defined chemical engineering processes possible.

**Figure 2:**
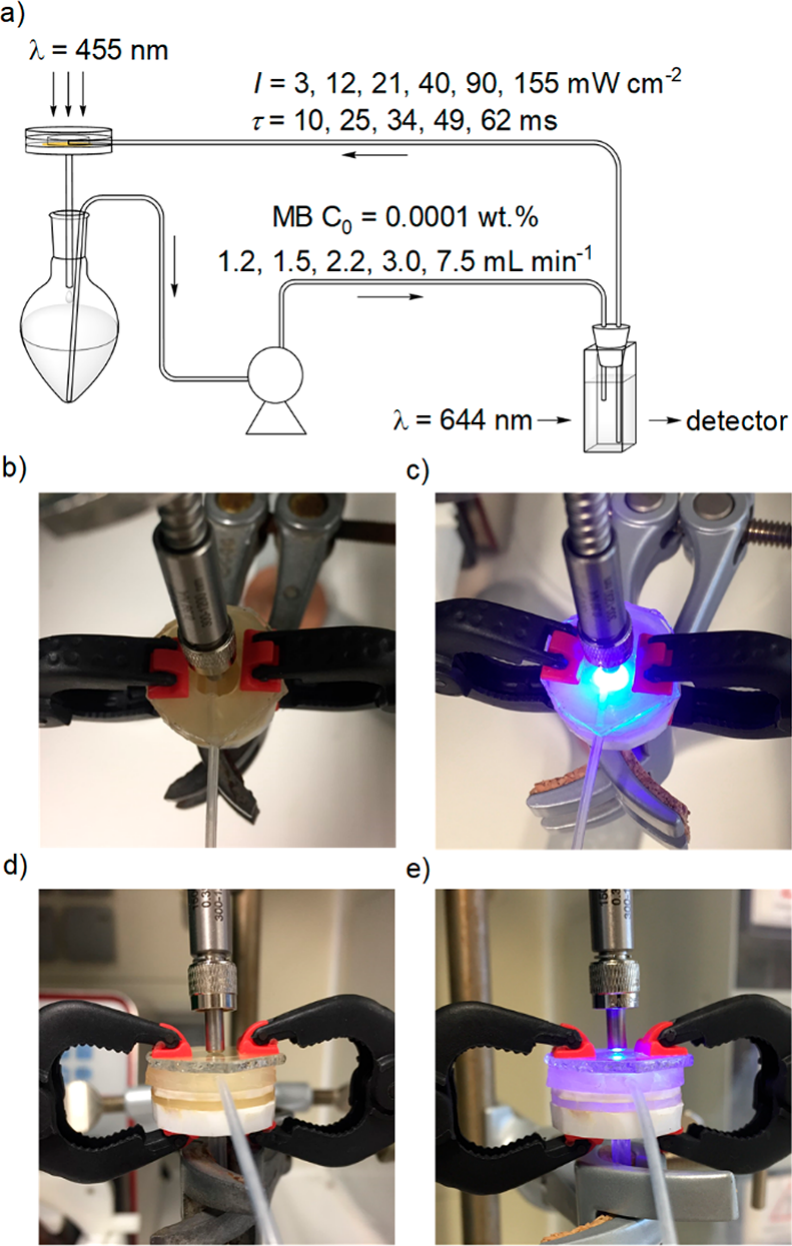
(a) Schematic diagram of the flow photoreactor incorporating a CNN membrane and some key process parameters. Top view (b, c) and side view (d, e) of the CNN membrane holder without and with light irradiation, respectively.

**Figure 3:**
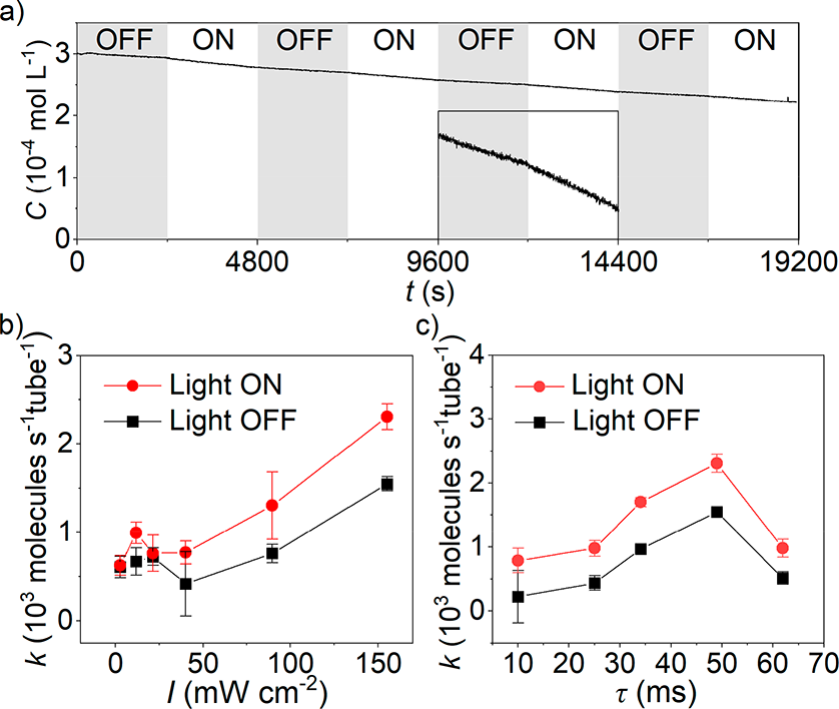
(a) Decay curve of MB in the flow reactor with the CNN membrane as the photocatalyst. Inset is the magnified area of the decay curve in the third dark/light cycle. (b) MB degradation rates (mean ± SD, *n* = 3) under light irradiation (different optical power) and in the subsequent dark cycle at τ = 49 ms and (c) with different residence time under light irradiation (155 mW cm^–2^) and in the subsequent dark cycle.

To evaluate the performance of the CNN membrane as a free-standing photocatalyst, degradation of methylene blue (MB) was chosen as a model reaction, because the progress could be easily monitored using absorption spectroscopy. This reaction was implemented in a circular flow reactor (Figure 2). Figure 3a shows the MB absorption decay curve under the optical power of 155 mW cm^–2^ and a flow rate of 1.5 mL min^–1^. The curve displays noticeable steps (see the magnified area in the inset of Figure 3a) where the degradation of MB proceeded at a higher rate under light irradiation than that in the dark. The decrease of MB concentration in the dark is explained by adsorption of the positively charged phenothiazinium cation at the negatively charged surface of carbon nitride (measured ζ-potential is −22.4 ± 1.3 mV). The influence of the electrolyte composition and ionic strength is given in Supplementary Discussion 1.

The CNN membrane is composed of densely packed, highly regular nanotubes, which can be simplified to be spatially isolated nanoreactors. This offers an opportunity to quantify the photocatalytic performance of carbon nitride. Thus the number of converted MB molecules per second per nanotube was used to describe the rate of degradation (*k*) and may serve as a quantitative descriptor of the membrane efficiency. With this quantification method, the dependence of the MB degradation behavior on the optical power (*I*) of incident light was studied at a flow rate of 1.5 mL min^–1^ (Figure S10). The reaction conditions used in this work and some other reports are summarized in Table S2. The degradation of MB proceeded at a higher rate with a higher optical power of incident light (Figure 3b). In this study, when the maximum *I* (155 mW cm^–2^) was applied, a highest degradation rate of 2308 ± 145 molecules s^–1^ tube^–1^ was obtained. The MB degradation rate indeed depends on *I*, which proves that the primary driver for this reaction is the amount of harvested photons that induce photogenerated electrons and holes to participate in the redox reactions. Dependence of *k* in the dark cycle *versus I* in the preceding light cycle could also be rationalized by the accumulation of electrons in the CNN membrane driven by irradiation with visible light.^57^

Residence time or contact time (τ) is a crucial parameter in flow chemistry, which is determined by the flow rate of the reagent stream and the volume of the reactor active zone (in our case, the confined space within carbon nitride nanotubes). We, therefore, optimized the MB degradation rate *via* control of residence time, which was realized by different flow rates (Figure S11). Figure 3c shows that the MB degradation rate increases with the residence time and reaches an optimum value of 2308 ± 145 molecules s^–1^ tube^–1^ at τ = 49 ms (corresponding to a flow rate of 1.5 mL min^–1^). It has been reported that TiO_2_ can reduce MB to the *leuco*-form at the expense of water as a sacrificial electron donor.^58,59^ Due to favorable positions of the CB and VB in the CNN membrane (Figure S12), such process might also be operative in our case, which explains the optimum of the residence time (Supplementary Discussion 2). Our conclusion is further supported by the experiment under anaerobic conditions (Figure S13). Thus, the rate of methylene blue degradation is higher under O_2_-free conditions, implying that reduction of oxygen, possibly to H_2_O_2_, competes with the reduction of MB to the *leuco*-form. The rate of MB degradation is also higher when the reaction was conducted in the presence of triethanolamine, a standard sacrificial electron donor in photocatalysis.

Finally, electronic transitions observed as the additional bands in the UV–vis absorption spectrum of the CNN membrane could be activated by green photons (λ = 530 nm) (Figure S14, Supplementary Discussion 3). Degradation of MB was also observed under red light (λ = 625 nm), which stems, at least partially, from the direct excitation of MB (Figure S14).

In spite of the low concentration, an overall, convoluted reaction time of about 50 ms for the photochemical processes is obviously extremely short and efficient and underlines impressively the validity of the nanoconfined catalysis concept. The above findings inspired examination of the feasibility of using the CNN membrane for confined organic synthesis, where indeed reaction cascades can by simpler. This was realized by conducting oxidation of amines into imines, which are important synthetic intermediates of medicines or biologically active nitrogen-containing organic compounds.^60,61^

**Figure 4:**
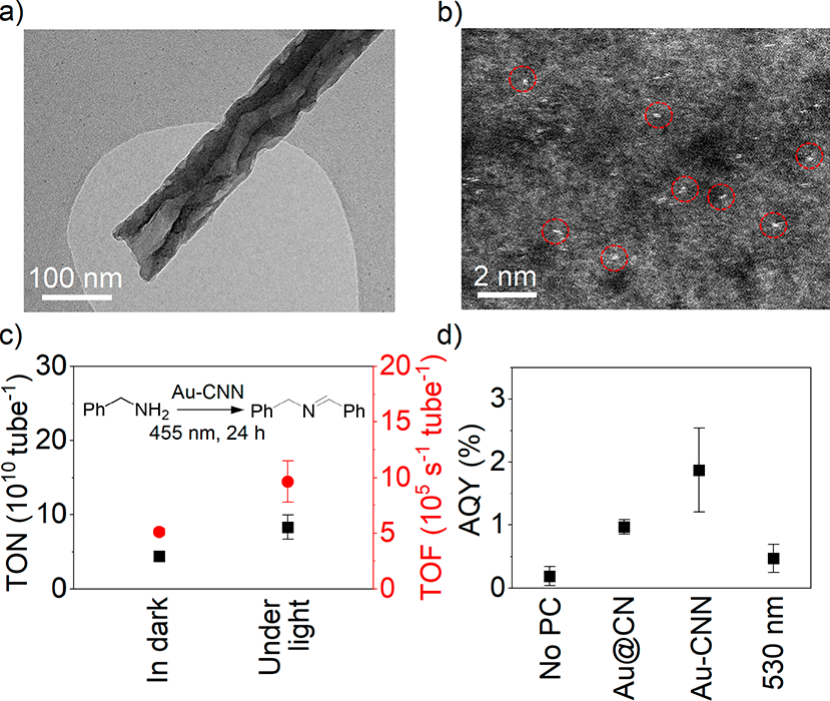
(a) TEM image of the single Au-CNN. (b) High-magnification HAADF-STEM image of the inner surface of the Au-CNN. Au single species are marked with red circles. TON (in black), TOF (in red) (c), and AQY (d) of benzylamine oxidation under different reaction conditions. Mean ± SD (*n* = 3). PC stands for photocatalyst.

For these experiments, a Au-modified CNN (Au-CNN) membrane was employed to catalyze the selective photooxidation reaction. A simple impregnation method was used to generate Au single sites that are well dispersed on the inner surface of carbon nitride nanotubes (Figure 4a,b, Supplementary Discussion 4).^62^ Benzylamine was selected as the substrate; molecular oxygen with a dissolved concentration of about 6.9 μg mL^–1^ was used as an acceptor of electrons and protons. The reaction was performed in a flow reactor; a photo of the setup is displayed in Figure S15. The calculation methods of turnover number (TON), turnover frequency (TOF), and apparent quantum yield (AQY) are described in the Supporting Information. The results are displayed in Figure 4c and d, and the data are summarized in Table S3, entries 1–4.

In the experiment under blue light irradiation (455 nm) with the Au-CNN membrane as the photocatalyst (entry 1, Table S3), the conversion efficiency of the reaction expressed as TON and TOF of a single carbon nitride nanotube for 24 h continuous operation is (8.31 ± 1.61) × 10^10^ and (9.63 ± 1.87) × 10^5^ s^–1^, respectively, with a high selectivity to give *N*-benzylidene benzylamine as the only product (Figure S16).^63^ To examine the confinement effect on the conversion of benzylamine, Au-deposited carbon nitride powder (Au@CN) (5 mg) instead of the Au-CNN membrane was used to perform the reaction (entry 4, Table S3). The results reveal that the AQY of the reaction in the bulk carbon nitride system was 1.0 ± 0.1%, which was only half that confined in carbon nitride nanotubes (1.9 ± 0.7%). Here we use AQY to evaluate the performance of the cases in the absence of the Au-CNN membrane. The conversion of the dark reaction was subtracted from the AQY. It was found that the oxidative coupling also occurred in the absence of light irradiation (Table S3, entry 3), but with a lower TOF of (5.12 ± 0.3) × 10^5^ s^–1^. This is probably due to the self-coupling between benzylamine molecules in flow mode where O_2_ was easily accessible. The presence of only light irradiation (entry 2) gave an AQY of 0.2 ± 0.1%. As in the MB photocatalytic degradation experiment, the Au-CNN membrane enables oxidation of benzylamine under green light; an AQY of 0.5 ± 0.2% was obtained in this case (Figure 4d). Under irradiation with 625 nm the AQY is close to the standard deviation of the measurements.

The scope of the substrates has been extended to benzylamines substituted in the aromatic ring (Table 1). Encouraged by the efficiency of the coupling of benzylamine and the ease of product separation, we showed application of imines as the aza-dienophiles in the reaction with an electron-rich diene, in which the corresponding piperidine-4-one has been obtained with 18% yield (Scheme S1). The set of experiments demonstrate a synthetic utility of the carbon nitride membrane in organic photoredox transformations.

**Table 1:**
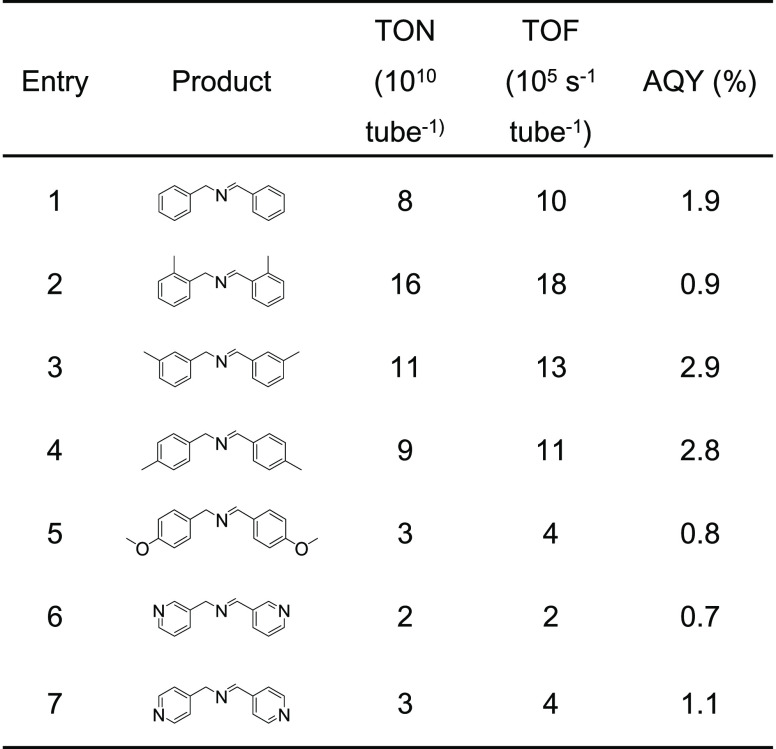
Photocatalytic Oxidation of a Series of Benzylic Amines with the Au-CNN Membrane^a^

a Reaction conditions: amine 2 mL; 455 nm; flow rate 1 mL min^–1^; in air; 24 h. The conversion of the dark reaction was subtracted from the AQY.

Spin-trapping experiments with 2,2,6,6-tetramethylpiperidine (TEMP) as the reagent for singlet oxygen (^1^O_2_) detection and 5,5-dimethyl-1-pyrroline-*N*-oxide (DMPO) for superoxide radical revealed that ^1^O_2_ is the main reactive oxygen species in the oxidation of benzylic amines (Figure S17).

As a preliminary conclusion, below we explain how the confinement effect in the CNN membrane affects the performance of the material in photocatalysis. The importance of the internal electric field, which among other stimuli may be induced either by band bending at the interface between the semiconductor and electrolyte or by polar surface terminations, in semiconductor photocatalysis is well-acknowledged.^55^ The profile of the electric field decay with the distance induced by the polar surface of the semiconductor in opened and confined environments is different. Unlike the open system, in which electric potential at the interface between semiconductor and electrolyte decays exponentially and approaches zero in the bulk of solution, in the pore, the electric potential decays toward the center and reaches a finite value (Figure S18).^64,65^ Therefore, in the pores, the molecules are confined in a strong electric field. We estimate that in the middle of the CN nanotube the electric field gradient is *ca*. 10^5^ V m^–1^ (Supplementary Discussion 1). Such an internal electric field has a dual effect. First, in the studied photocatalytic reactions, it induces polarization of molecules, which in turn enhances their reactivity. The profound impact of the electric field, in particular an oriented external electric field, on the reactivity of molecules has been studied theoretically and confirmed experimentally.^66−70^ Second, the concentration of the internal electric field in the nanotube induced by the confinement effect facilitates separation of the photogenerated charge carriers and extends their lifetime (Figure 1f). As a result, photogenerated electrons and holes are utilized more efficiently, which leads to a higher AQY (Figure 4d). Finally, efficient utilization of the confinement effect is tightly linked to the reactor design. Contrary to batch reactors, in which at any time point only a tiny fraction of the reaction mixture is confined, for instance, in the voids of mesoporous materials, in the flow reactor, such as the CNN membrane, the whole reaction mixture volume is confined. In the opened system, only a relatively small fraction of molecules is located in close proximity to the surface of the semiconductor and therefore experience an electric field induced by the negatively charged surface of carbon nitride. The vast majority of molecules is located in the bulk. We believe that such efficient utilization of confined space in the flow reactor is responsible for the very high TOF of the CNN membrane in photocatalysis compared to some of the reported photocatalytic systems (Table S4).

The last aspect leads to the final point of discussion related to changes in fluid transport through the CNN membrane operating as a flow photoreactor compared to opened systems. It is well established that the transport of fluids in confined conditions differs fundamentally from that in the bulk due to the interaction between the fluid molecules and the solid wall,^71^ but also due to a possible size dependence of viscosity. Thus, it would be interesting to quantify the changes in our confined fluids, which may help us to understand how confinement alters the behavior of molecules in chemical reactions. Numerous studies have demonstrated a pressure-driven ultrafast flow through the interior of CNTs and graphene nanochannels, far exceeding that predicted by continuum hydrodynamic theories, both by molecular dynamics simulations and experimental investigations.^72,73^ However, the flow enhancement behaviors of fluids in 40 nm diameter carbon nitride nanotubes would nicely complement these data, as we are with our system in a more mesoscopic range and thereby potentially closer to the transition from bulk to confined behavior; in other words, is quantum transport really extended to the length scale of 40 nm?

**Figure 5:**
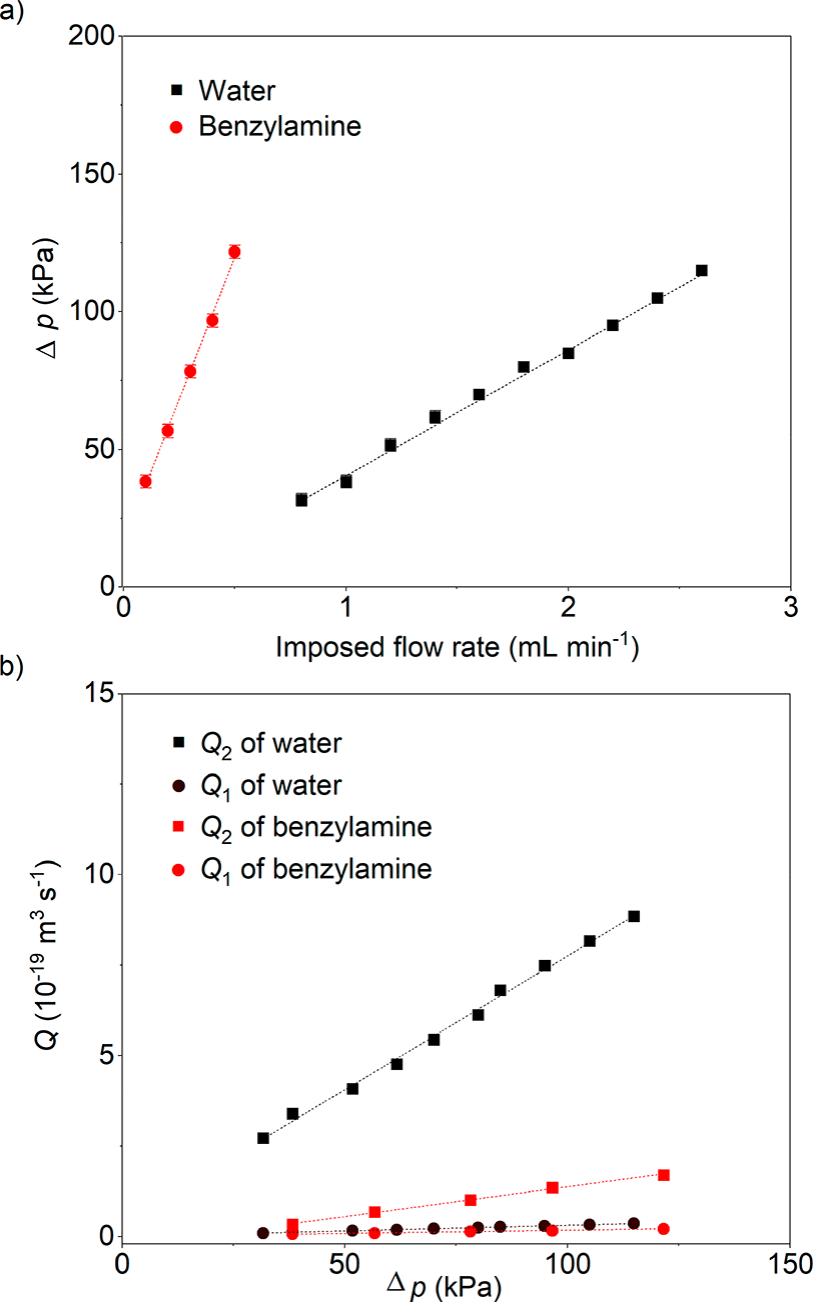
(a) Plot of Δ*p* against imposed flow rate of water and benzylamine through the CNN membrane. Mean ± SD (*n* = 3). Dashed lines are linear fits with *R*^2^ > 0.99. (b) *Q*_1_ and *Q*_2_ of water and benzylamine through the CNN membrane under a range of Δ*p*. Dashed lines are the linear fits with *R*^2^ > 0.99.

The CNN membrane formed by highly dense and vertically aligned carbon nitride nanotubes serves as a perfect platform to study the nanoscale fluid transport quantitatively. To determine the flow enhancement, we measured the pressure drop (Δ*p*) of fluids passing through the CNN membrane at a range of imposed flow rates (*Q*_0_) (Figure S19). A positive linear relationship between Δ*p* and *Q*_0_ was observed for both water and benzylamine (Figure 5a). This excludes interface yield stress and secured Newtonian flow through the nanosized capillary. The theoretical bulk flow rate through a single carbon nitride nanotube (*Q*_1_) can be predicted from the classical Hagen–Poiseuille equation (eq 3):
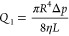
3where *Q*_1_ is the theoretical flow rate through a single nanotube, m^3^ s^–1^; *R* is the inner radius of nanotube, m; Δ*p* is the pressure drop, Pa; η is the dynamic viscosity of the fluid (1.002 × 10^–3^ Pa s for water and 1.780 × 10^–3^ Pa s for benzylamine); and *L* is the length of the nanotube, m. Details of *Q*_2_ calculation are given in the Supporting Information.

A significant flow enhancement was observed for water and benzylamine in comparison to the theoretical calculation (Figure 5b). The average enhancement factor (ε) for water was calculated to be 25 ± 1. This indicates a flow about 25 times faster than predicted from bulk properties through the 40 nm diameter carbon nitride nanotube. The result is close to the experimentally observed ε of 28–45 for water flowing through 44 nm CNTs in the previous study,^28,73−77^ but is very much less than that reported through <10 nm diameter CNTs.^74,75^ Benzylamine showed a relatively smaller flow enhancement with an ε of 7 ± 1. We attribute that to water being an interacting, structured solvent where confinement effects are expected to have a higher impact. Note that the measurements under light irradiation did not give any dependence of ε on the light intensity involved.

Flow enhancement could also be conventionally described using the notation of a slip length (*l*_s_), which is a mathematical extra tube radius required to give zero velocity at a hypothetical tube wall (see Supporting Information for details of *l*_s_ calculation). The calculated *l*_s_ for water (123 ± 7 nm) and benzylamine (30 ± 6 nm) are so far away from the measured tube radius that we can confirm a decrease in viscosity and thus less friction in the nanotubes.^74,78,79^

What interests us most are the changes in chemistry that come with the physics imposed by nanoconfinement. Here several factors can be possibly responsible for the powerful catalysis observed in carbon nitride nanotubes. It is accepted that oxygen diffusion plays an important role in the catalytic oxidation of organic compounds.^80^ According to the Stokes–Einstein equation (eq 4),^81^
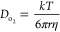
4where *D*_O2_ is the oxygen diffusion coefficient, cm^2^ s^–1^; *k* is the Boltzmann constant, kg cm^2^ s^–2^ K^–1^; *T* is the absolute temperature, K; *r* is the radius of the O_2_ molecule, cm; and η is the dynamic viscosity of the liquid, kg s^–1^ cm^–1^. The oxygen diffusion coefficient in a liquid is inversely proportional to its viscosity. In our case, the decreased viscosity of fluids due to nanoconfinement can improve the oxygen mobility in the fluids, which may lead to a higher catalytic activity or even selectivity.^82^

Apart from a nearly frictionless liquid/solid interface, the ordering of fluid molecules at the confining walls could also play a role in the ultrafast flow under spatial confinement.^83^ Such ordering induced by 1D spatial confinement was known to be 1D templating effect and was shown experimentally to enhance the activity and selectivity of catalytic processes, *e*.*g*., dehydrogenative C–C coupling reactions, by decreasing the activation energy.^84,85^ Indeed, definition of an entropy under such conditions is difficult, as the partition function is certainly much smaller, and entropy thereby plays a less decisive role in the free reaction enthalpy. This in general favors controlled, energy-driven reactions and disfavors uncontrolled, entropy-driven reactions; that is, the reaction goes to more organized, more complex reaction states and discriminates for stable compounds.

## Conclusions

In summary, we demonstrated that the flow-through CNN membrane with 1D nanochannels can be employed as a photocatalytically active nanoreactor for confined organic conversions. The membrane displayed an excellent performance in MB degradation with a rate of 2308 ± 145 molecules s^–1^ in a single carbon nitride nanotube. The Au-deposited CNN membrane allowed for amine oxidation in a single nanotube with an AQY up to 1.9 ± 0.7%, which is notably higher than that obtained with bulk carbon nitride. We propose that the highly confined environment of this nanoreactor can result in significant changes to chemistry in comparison to that observed in bulk systems, which plays a critical role in the enhanced catalytic activity. At the same time, the concave surface of the CN nanotube is an integral part of the nanophotoreactor and confinement effect. A concave surface maintains an internal electric field induced by the polar surface of the carbon nitride inside the nanotube, which has multiple positive effects on the performance of the material when it is assembled into a membrane: (1) polarization of reagent molecules; (2) extension of lifetime of the photogenerated charge carriers; (3) more efficient utilization of the confined effect using flow technology compared to the reaction in batch.

Quantification of the dynamics of the confined fluids reveals an enhanced flow compared to predictions based on bulk behavior. This highlights how confinement can be used to realize highly active and selective catalysis by changing otherwise fundamental physical properties, such as viscosity or even the more primary partition function.

Despite the quantity of work already carried out on confined catalysis, the challenge remained to develop tools that are viable for larger scale industrial synthesis. Given the efficacy of the CNN membrane in organic synthesis as demonstrated by the examples in this paper working already on the mL min^–1^ level, together with its attractive advantages such as simple product separation and catalyst recovery, semitransparency, low back pressure, and the possibility to quantify and optimize performance within a single nanoreactor, this tool is expected to be relevant for many other chemical transformations. Exemplarily, our next steps will apply 1D carbon nitride nanochannels that are dimensionally comparable to small biomolecules, which may open selectivity options for enzymes and protein functionalization beyond our current understanding.

## Methods

### Fabrication of the CNN Membrane

The CNN membrane was fabricated with a vapor-deposition polymerization (VDP) method described previously.^45^ First, the commercial AAO membrane was washed with ethanol and deionized water, then dried by purging with nitrogen. The AAO membrane and melamine (0.5 g) were placed into the bottom of a glass test tube. The tube was heated in an oven at 773 K for 4 h with a heating rate of 10 K min^–1^. After the temperature naturally cooled to room temperature, the CNN/AAO membrane was obtained. The carbon nitride powder at the bottom of the glass test tube was collected for comparative studies. To remove the AAO template, the CNN/AAO membrane was immersed in 1 M HCl for 72 h, washed with deionized water, and then dried at 60 °C in an oven.

### Fabrication of the Au-Loaded CNN Membrane

Typically, the CNN membrane was immersed in water (15 mL) with slow stirring. Then, different amounts of HAuCl_4_ aqueous solution (0.02 M) were slowly added into the above mixture. For the single-site Au catalyst used in this work, 0.10 mL of HAuCl_4_ aqueous solution was added. After stirring at room temperature for 7 h, the Au-CNN membrane was collected by tweezers, washed with water, and dried under vacuum at 60 °C overnight, followed by reducing under an Ar/H_2_ atmosphere at 623 K for 1 h with a heating rate of 10 K min^–1^, giving a 0.2 wt % Au loaded CNN (Au-CNN) membrane. Au-loaded carbon nitride powder (Au@CN) was prepared with the same procedure except that the CNN membrane was replaced with carbon nitride powder.

### Method of Photocatalytic MB Degradation with the CNN Membrane

A MB aqueous solution (0.05 wt %, 0.2 mL) was diluted with deionized water in a volumetric flask to obtain a MB stock solution (0.0001 wt %, 100 mL). For the photocatalytic experiment, the MB stock solution (4 mL) was pumped (diaphragm metering pump, Grundfos DDE 6-10) through the CNN membrane for 320 min under blue LED irradiation (455 nm). The light was turned on and off every 40 min. The absorbance of the MB solution at 664 nm in the flow cell was measured with UV–vis spectroscopy every 3 s.

A photo of the setup is shown in Figure S6. The CNN membrane was supported on a holder (Figure S7). The flow was confined by a silicone rubber gasket to pass through the central area with a 7 mm diameter, which is considered as the effective area of the membrane. The vertically aligned nanotubes allow for a flow that is only parallel to the light. The dissolved oxygen concentration in the MB solution approximates the equilibrium oxygen concentration in water under ambient conditions (8 μg mL^–1^). The calibration curve used for the conversion of absorbance (a.u.) at 664 nm to concentration (mol L^–1^) is displayed in Figure S8.

To confirm that this is a photocatalytic process catalyzed by carbon nitride, the reaction was performed in the flow reactor in the absence of the CNN membrane. As shown in Figure S9, a negligible MB degradation behavior was observed, demonstrating that the reaction could hardly occur without the CNN membrane acting as the photocatalyst.

### Method of Photocatalytic Oxidation of Amine with the Au-CNN Membrane

Benzylamine (2 mL) was pumped (peristaltic pump, Ismatec MCP-Process IP65) through the Au-CNN membrane at a flow rate of 1 mL min^–1^ for 24 h under blue LED irradiation (455 nm). After the irradiation the reaction mixture was collected and analyzed by ^1^H NMR using 1,3,5-trimethoxybenzene as internal standard. Repeating experiments were conducted three times.

In control experiments, the procedure was as described above, but (1) without the Au-CNN membrane, (2) without light irradiation, and (3) Au@CN spread over filter paper was used instead of the Au-CNN membrane.

### Method of Aza-Diels–Alder Reaction

Imine (∼46 mg) was separated from the reaction mixture of benzylamine oxidation with the Au-CNN membrane by distillation under vacuum at 60 °C. 1-Methoxy-3-trimethylsiloxy-1,3-butadiene (21 mg) was added to the solution of the imine in MeOH (2 mL), which was stirred for 1 h. A second portion of 1-methoxy-3-trimethylsiloxy-1,3-butadiene (21 mg) was added, and the stirring was continued for 2 days. The reaction mixture was quenched with HCl (0.1 mL, 37 wt % in water) and stirred for 2 h. The product was extracted with diethyl ether, dried over Na_2_SO_4_, concentrated under vacuum (50 °C, 30 mbar), and analyzed by ^1^H NMR with 1,3,5-trimethoxylbenzene as internal standard.

### Method of EPR Measurement for O_2_^•–^ and ^1^O_2_ Detection

For O_2_^•–^ detection, a solution of DMPO (8.5 μL) in ethanol (3 mL) was pumped through the Au-CNN membrane at a flow rate of 1 mL min^–1^ for 18 h under blue LED irradiation (455 nm). After the irradiation the solution was collected and analyzed by EPR spectroscopy. For ^1^O_2_ detection, the procedure was repeated with a solution of TEMP (16.7 μL) in ethanol (3 mL).

The EPR study was conducted on a Bruker EMXnano benchtop X-band EPR spectrometer. The following settings were used for the spectra acquisition: center field 3444.05 G, sweep width 200 G, receiver gain 40 dB, modulation amplitude 1.000 G, number of scans 16, microwave attenuation 25 dB. A capillary (IntraMark, volume 50 μL, purchased from BRAND GMBH + Co. KG) was sealed in the flame of a gas burner from one side. The capillary was charged with the solution (40 μL) collected after irradiation. The capillary was placed into an EPR tube (i.d. 3 mm, o.d. 4 mm, length 250 mm). The EPR spectrum was acquired in the dark at room temperature.

### Method of Pressure Measurement over the CNN Membrane

A syringe pump (KD Scientific 789100 B) with a 20 mL capacity plastic syringe was used to drive fluids to flow through the CNN membrane. The pressure above the membrane was monitored by a pressure meter (Fisherbrand PS100-50BAR) located between the syringe pump and the membrane holder. Pressure was recorded at each flow rate once readings were stable over a minimum period of 60 s. Reading was repeated three times at intervals of every 60 s. The pressure behind the membrane was approximately 1 atm. The pressure meter reads 0 at 1 atm; therefore the observed pressure was equal to the pressure drop (Δ*p*).

### Method of Zeta-Potential Measurement

For zeta-potential measurement, a sample suspension containing 1 mg of sample in 2 mL of deionized water and MB aqueous solution (0.0001 wt %), respectively, was prepared and sonicated for 1 h before use. The capillary zeta cell (Mavern DTS1060) was charged with the suspension and sealed with stoppers. The zeta potential was recorded on a Zetasizer Nano ZS zeta-potential analyzer with a hold time at 120 s.

### Method of Fluorescence Lifetime Measurement

TR-PL spectra were recorded on a fluorescence lifetime spectrometer (Fluo Time 250, PicoQuant) equipped with a PDL 800-D picosecond pulsed diode laser drive. The decay curves were fitted using a nonlinear method with a multicomponent decay law given by *I*(*t*) = a_1_ exp(−*t*/τ_1_) + *a*_2_ exp(−*t*/τ_2_) + *a*_1_ exp(−*t*/τ_3_).

A sample suspension containing 1 mg of catalyst in 2 mL of solvent was prepared and sonicated for 1 h before use. The TR-PL spectra were obtained with λ_exc_ = 375 nm and λ_em_ = 500 nm. The following settings were used for the spectra acquisition: laser frequency 40 MHz, emission monochromator bandwidth 10 nm.

### Characterization

Powder X-ray diffraction (PXRD) patterns were recorded on a Bruker D8 Advance instrument with Cu Kα radiation. SEM images were obtained on a JSM-7500F (JEOL) at an accelerating voltage of 3 kV. TEM images were obtained on a double-corrected JEOL ARM200 at an acceleration voltage of 200 kV and an emission of 10 μA. Optical absorbance spectra were measured on a Shimadzu UV 2600. PL spectra were recorded on an FP-8300 fluorescence spectrometer. ^1^H NMR spectra were recorded on an Agilent 400 MHz spectrometer using the residual signal of chloroform in CDCl_3_ (7.26 ppm) as a reference and 1,3,5-trimethoxybenzene as internal standard.

### Electrochemical Measurement

The flat-band potential (*E*_fb_) measurement was carried out with an Arbin electrochemical testing station (Arbin Instrument) in a standard three-electrode quartz cell. The working electrode was prepared as follows: 2 mg of sample was suspended in 0.2 mL of deionized water containing 0.02 mL of a 5 wt % Nafion D-520 dispersion, and the mixture was then dispersed by ultrasonication and spread onto an FTO glass. After being dried naturally, the FTO glass was heated at 120 °C for 1 h. The prepared thin film was employed as a working electrode, with a platinum plate as a counter electrode and Ag/AgCl as a reference electrode (3 M KCl). A 0.5 M Na_2_SO_4_ aqueous solution was used as an electrolyte. The measurement was carried out at a frequency of 10 kHz in a potential range from −1.0 to 0.4 V *versus* Ag/AgCl. The measured potentials *versus* Ag/AgCl were converted to the RHE scale according to the Nernst equation:
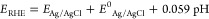
5where *E*_RHE_ is the converted potential *versus* RHE, *E*^o^_Ag/AgCl_ = 0.1976 at 25 °C, and *E*_Ag/AgCl_ is the experimentally measured potential against the Ag/AgCl reference.

## Supporting Information Available

Full details of the experimental and computational protocols, supplementary discussion, complementary analytical data (PDF)
