# Supplementary material for: Enhanced Organic Photocatalysis in Confined Flow through a Carbon Nitride Nanotube Membrane with Conversions in the Millisecond Regime
Source: ACS Nano. 2021 Apr 6;15(4):6551–61. doi: 10.1021/acsnano.0c09661 (PMC8155341; doi:10.1021/acsnano.0c09661)
Supplement: Supplementary file 1 — nn0c09661_si_001.pdf [file nn0c09661_si_001.pdf]

Supporting Information

Enhanced Organic Photocatalysis in Confined Flow through a Carbon Nitride Nanotube Membrane with Conversions in the Millisecond Regime

*Yajun Zou, Kai Xiao, Qing Qin, Jian-Wen Shi, Tobias Heil, Yevheniia Markushyna, Lei Jiang, Markus Antonietti, and Aleksandr Savateev**

Experimental section

Materials

Melamine (> 98.0%) was purchased from Sigma-Aldrich. The target 60-μm-thick anodic aluminium oxide (AAO) membrane with pore width 84 ± 16 nm was purchased from Heifei Puyuan Nano, China. Glass test tube for vapor-deposition polymerization (VDP) was purchased from Merck Millipore. Methylene blue (MB) (0.05 wt.% in water solution) was purchased from Sigma-Aldrich. $HAuCl_4 \cdot xH_2O$ (≥ 49% Au basis) was purchased from Sigma-Aldrich. 1,3,5-trimethoxybenzene (> 98%) was purchased from TCI. Benzylamine (99.0%) was purchased from Sigma-Aldrich. (4-Methoxyphenyl)methanamine (98%) was purchased from Sigma-Aldrich. 2-Methylbenzylamine (96%) was purchased from Sigma-Aldrich. 3-Methylbenzylamine (98%) was purchased from Sigma-Aldrich. 4-Methylbenzylamine (97%) was purchased from Sigma-Aldrich. 3-Picolylamine (≥ 99%) was purchased from Sigma-Aldrich. 4-(Aminomethyl)pyridine (98%) was purchased from Sigma-Aldrich. 5,5-Dimethyl-1-pyrroline-N-oxide (DMPO) (≥ 98%) was purchased from Enzo. 2,2,6,6-Tetramethylpiperidine (TEMP) (> 98%) was purchased from Alfa Aesar. 1-Methoxy-3-trimethylsiloxy-1,3-butadiene (95%) was purchased from Sigma-Aldrich. Methanol was purchased from Merck. Ethanol was purchased from Merck. HCl (37%) was purchased from Acros Organics. Diethyl ether was purchased from VWR chemicals. Nafion D-520 dispersion (5 wt. % in mixture of lower aliphatic alcohols and water, contains 45% water) was purchased from Sigma-Aldrich. Na_2SO_4 (anhydrous, 99.0-100.5%) was purchased from Sigma-Aldrich. Fluorine doped tin oxide (FTO) coated glass was purchased from Sigma-Aldrich. Capillary (IntraMark, volume 50 μL) was purchased from BRAND GMBH + CO KG.

Light source

A light emitting diode (LED, M455F3 purchased from ThorLabs) module with emission maximum at 455 nm coupled with the optical fiber and controlled by the driver (DC2200, purchased from ThorLabs) was used in the photocatalytic tests and spin-trapping experiment. The light intensity was measured with an integrating sphere photodiode power sensor (S142C purchased from ThorLabs).

Membrane holder assembly

A draft of the home-made membrane holder is shown in the Figure S7. In this configuration FEP tubing 1/16'' was used as inlet, while FEP tubing 1/8'' was used as outlet. Tube clamps with screw conductive (Carl Roth, ROTILABO®), FEP ferrules (1/16'', Upchurch Scientific (Part No. UPP-200N) and 1/8'', Upchurch Scientific (Part No. UPP-300N)), FEP nuts (1/16'', Upchurch Scientific (Part No. UPP-245) and 1/8'', Upchurch Scientific (Part No. UPP-345)) and union assemblies (Tefzel (ETFE), Upchurch Scientific (Part No. UPP-630)) were used to connect the membrane holder assembly to the pump inlet and outlet (and flow-through cuvette for MB degradation experiments).

Calculation

Calculation of average rate of MB degradation

The average rate of MB degradation (k) was calculated as

$$k = \frac{r \times V \times N_A}{n_{tube}}$$

where k – the average rate of MB degradation, s^{-1} $tube^{-1}$; r – the average rate of MB degradation obtained by linear fitting of the decay curve during each light on and off cycle, mol L^{-1} s^{-1}; V – the volume of MB solution in the reactor, L; N_A–the Avogadro constant, 6.02×10^{23} mol^{-1}; n_{tube} – the number of carbon nitride nanotubes on the effective area of the CNN membrane, which was calculated as

$$n_{tube} = \frac{S_{membrane}}{S_{tube}} = \frac{d^2_{membrane}}{d^2_{tube}} = 4.9 \times 10^{10}$$

where n_{tube} – the number of carbon nitride nanotubes on the effective area of the CNN membrane; $S_{membrane}$ – the effective area of the CNN membrane, mm^2; S_{tube} – the cross-sectional area of carbon nitride nanotube, mm^2; $d_{membrane}$ – the diameter of the effective area of CNN membrane, mm; d_{tube} – the external diameter of carbon nitride nanotube, mm.

Calculation of residence time

The residence time (τ) was calculated as

$$\tau = \frac{V}{v} = \frac{\frac{\pi \times D^2}{4} \times L \times n_{tube}}{v}$$

where τ – the residence time, ms; V – the cumulative volume of cavity in the CNN membrane, cm^3; v – the flow rate of MB solution, cm^3 ms^{-1}; D – the inner diameter of carbon nitride nanotube, cm; L – the thickness of the CNN membrane, cm; n_{tube} – the number of carbon nitride nanotubes on the effective area of the CNN membrane.

Calculation of turnover number and turnover frequency of single carbon nitride nanotube

The turnover number (TON) of single carbon nitride nanotube was calculated as

$$TON = \frac{n_{\text{amine}}}{n_{\text{tube}}} = \frac{\frac{d \times V}{M} \times (2 \times (w_1 - w_2)) \times N_A}{n_{\text{tube}}}$$

where TON – the turnover number; n_{amine} – the number of converted amine molecules; n_{tube} – the number of carbon nitride nanotubes on the effective area of the CNN membrane; d – the density of amine, g mL^{-1}; V – the volume of amine, mL; M – the molar mass of amine, g mol^{-1}; w_1 – the imine content after the reaction; w_2 – the imine content before the reaction (0.75%); N_A – the Avogadro constant, 6.02×10^{23} mol^{-1}.

The turnover frequency (TOF) of a single carbon nitride nanotube was calculated as

$$TOF = \frac{TON}{t}$$

where TOF – the turnover frequency, s^{-1}; TON – the turnover number; t – the duration of the experiment, s.

Calculation of apparent quantum yield of *N*-benzyl-1-phenylmethanimine

The apparent quantum yield (AQY) of imine was calculated as:

$$AQY = \frac{n_{\text{amine}}}{n_{\text{photon}}} \times 100\ \% = \frac{\frac{d \times V}{M} \times (2 \times (w_1 - w_3)) \times N_A}{I \times S_{membrane} \times t \times \frac{\lambda}{hc}} \times 100\ \%$$

where AQY – the apparent quantum yield of imine; n_{amine} – the number of converted amine molecules; n_{photon} – the number of incident photons; d – the density of amine, g mL^{-1}; V – the volume of amine, mL; M – the molar mass of amine, g mol^{-1}; w_1 – the imine content after the reaction; w_3 – the imine content after the dark reaction without catalyst (1.60%); N_A – the Avogadro constant, 6.02×10^{23} mol^{-1}; I – the light intensity, W cm^{-2}; S_{membrane} – the effective area of the CNN membrane, cm^2; t – the duration of the experiment, s; λ – the wavelength of incident light, m; h – the Planck's constant, 6.626×10^{-34} J s; c – the speed of light, 3×10^8 m s^{-1}.

Calculation of the actual flow rate through a single carbon nitride nanotube

The actual flow rate through a single carbon nitride nanotube (Q_2) was calculated as:

$$Q_2 = \frac{Q_0}{n_{\text{tube}}}$$

where Q_2 – the actual flow rate through a single carbon nitride nanotube, $m^3 s^{-1}$; Q_0 – the imposed flow rate, $m^3 s^{-1}$; n_{tube} – the number of carbon nitride nanotubes on the effective area of the CNN membrane.

Calculation of enhancement factor

The enhancement factor (ε) was calculated as:

$$\varepsilon = \frac{Q_2}{Q_1}$$

where ε – the enhancement factor; Q_2 – the actual flow rate through a single nanotube, $m^3 s^{-1}$; Q_1 – the theoretical flow rate through a single nanotube, $m^3 s^{-1}$.

Calculation of slip length

The slip length (l_s) was calculated as:

$$l_s = \frac{R}{4}\left(\frac{Q_2}{Q_1} - 1\right)$$

where l_s – the slip length, nm; R – the inner radius of nanotube, nm; Q_2 – the actual flow rate through a single nanotube, $m^3 s^{-1}$; Q_1 – the theoretical flow rate through a single nanotube, $m^3 s^{-1}$.

Supplementary discussion

Supplementary discussion 1.

In polar solvents, such as water, carbon nitride surface gains negative charge due to dissociation of surface hydroxyl groups possessing acidic character or adsorption of negatively charged ions from the solution.[2] Zeta-potential defines electric potential at the distance corresponding to Debye length (λ_D), which is calculated using the equation (1):

$$\lambda_D = \frac{1}{\sqrt{8 \cdot \pi \cdot l_B \cdot I}} \tag{1}$$

where l_B – is Bjerrum length, nm; I – ionic strength of the solution, mol L^{-1}.

Bjerrum length is defined by the equation (2):

$$l_B = \frac{e^2}{4 \cdot \pi \cdot \varepsilon_0 \cdot \varepsilon \cdot k_B \cdot T} \tag{2}$$

where e – elementary charge, $1.6 \cdot 10^{-19}$ C; ε_0 – vacuum permittivity, $8.85 \cdot 10^{-12}$ F m^{-1}; k_B – Boltzmann's constant, $1.38 \cdot 10^{-23}$ $J \cdot K^{-1}$; ε – dielectric constant of the medium; T – temperature, K.

Ionic strength is defined by the equation (3):

$$I = \frac{1}{2} \sum_{i=1}^{n} c_i z_i^2 \tag{3}$$

where c_i – molar concentration of ions i, mol L^{-1}; z_i – charge of ion i.

Using equations (1), (2) and (3) Debye length for pure water, aqueous methylene blue solution used in the photocatalytic experiments have been calculated and results are given in the Table S5.

Polar solvents facilitate ionization of surface functional groups and give shorter λ_D. Contrary non-polar solvents do not facilitate ionization of surface functional groups and give longer λ_D. However, it has been shown that even in non-polar solvents, nanoparticles, such as SiO_2, still possess high surface charge and characterized by relatively high zeta-potential.[3]

Comparison study of the CNN membrane and reference CN powder, shows that zeta-potential of reference carbon nitride is independent on presence or absence of methylene blue (Table S6). However, zeta-potential of the CNN membrane becomes less negative upon addition of methylene

blue. Such results indicate that absorption of phenothiazinium cation in the CN nanotube is significantly enhanced. Taking into account that both reference carbon nitride and the CNN membrane are made of the same material such enhancement is related to the confined environment.

Electric potential of the particle immersed into a polar solvent decays exponentially and approaches zero at infinite distance, while Debye length defines the distance at which electric potential decreases by e times (eq. 4):

$$\varphi = \varphi_0 e^{-\frac{r}{\lambda_D}} \quad (4)$$

where ϕ_0 – electric potential at carbon nitride surface, mV; r – distance from the surface, nm.

Taking into account measured zeta-potential, electric potential at zero distance (at carbon nitride surface) is calculated from the equation (5):

$$\varphi_0 = \zeta \cdot e \quad (5)$$

where ζ – zeta-potential, mV; e – Euler number, e = 2.72.

The results of φ_0 calculation are given in the Table S6.

Given that Debye length for all electrolytes used in this work, exceeds the internal diameter of the CN nanotube (d = 40 nm), electric field gradient (E) caused by drop of surface potential with the distance may be estimated using the linear equation (Table S6):

$$E = \frac{\varphi_c - \varphi_0}{\left(\frac{d}{2}\right)} \quad (6)$$

where φ_c – potential at the axis of the CN nanotube, i.e. at the distance 20 nm from the inner wall of the nanotube, V; φ_c – potential at the surface of the CN nanotube. Calculated using equation (4), V; d – inner diameter of the CN nanotube, m.

Supplementary discussion 2. Mechanism of methylene blue degradation.

Excitation of the CNN membrane followed by separation of charges gives electrons and holes with redox potentials of –0.75 V and +1.95 V *versus* NHE respectively. Due to more positive redox potential of holes compared to the redox potential of the reaction $H_2O - 4e^- = O_2 + 4H^+$ (+1.23 V *versus* NHE), the holes are quenched at the expense of water (Figure S12). At the same time, one-electron reduction of methylene blue gives $MB^•$ radical (E = –0.23 V *versus* NHE). The latter readily disproportionates into MB^+Cl^- and *leuco*-form of methylene blue (LMB), which does not possess absorption band at 664 nm.[4] As disproportionation requires addition of H^+ and Cl^- to the $MB^•$ as well as presence of a second molecule of $MB^•$, this reaction proceeds the most effectively in the region close to the surface of the heterogeneous photocatalyst in Helmholtz layer, where concentration of ions is the highest.

According to the mechanism of dye degradation (Figure S12), we propose that methylene blue is reduced to *leuco*-form at the expense of water as sacrificial donor of electrons and protons. Oxidation of water is accompanied by the formation of O_2, which is converted to singlet oxygen (Figure S17) as well as other potential reactive oxygen species.

Such reactive oxygen species facilitate oxidation of *leuco*-methylene blue to methylene blue (MB^+Cl^-) according to the reaction:

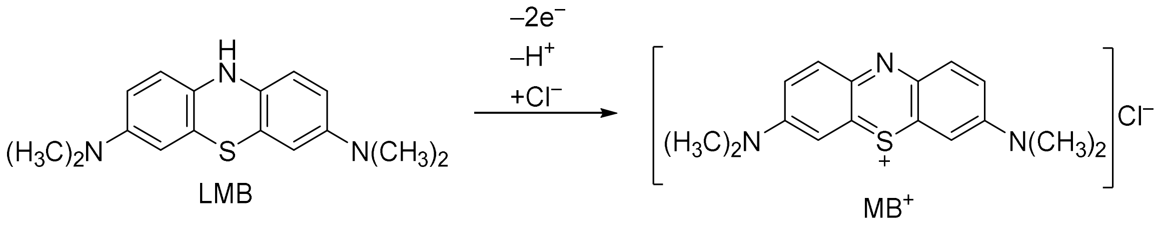

Redox potential of this reaction is E = +0.011 V *versus* NHE at pH = 7.[4] Therefore, once the residence time becomes long enough, recovery of methylene blue is facilitated. Therefore, concentration of methylene blue raises, which is registered as increase of the intensity of the absorption band at 664 nm and decrease of the degradation rate (*k*).

Supplementary discussion 3. Degradation of methylene blue using the CNN membrane as the flow photoreactor under blue (455 nm), green (530 nm) and red (625 nm) light.

At 455 nm absorption of MB is significantly lower compared to the absorption of the CNN membrane (due to $\pi - \pi^*$ transitions). Therefore, the enhanced rate of methylene blue degradation under blue light is due to the CNN membrane acting as the photocatalyst. Such conclusion is also in agreement with the fact that methylene blue does not degrade under blue light without the CNN membrane (Figure S9).

On the other hand, methylene blue itself possesses an absorption band with the maximum at 664 nm. Visible light induced photobleaching of MB under aerobic and anaerobic conditions proceeds *via* electron transfer between two molecules and formation of *leuco*-form (reduced form) of the dye.[5] Since absorption of light with λ = 625 nm by the CNN membrane is much lower, in our case, degradation of methylene blue under red light is apparently due to the photobleaching of the dye, while the role of the CNN membrane as the catalyst at this wavelength is only minor.

While methylene blue does not absorb significantly light with λ = 530 nm, clear enhancement of the degradation rate is seen under green light.

Supplementary discussion 4. CNN membrane modified with Au single atoms (Au-CNN membrane).

In our previous report on single-site Au loaded N-doped porous noble carbon for electrochemical reduction of nitrogen[6], we found that Au was nicely present as single species at a mass loading of 0.2 wt. %. Excessive amount of Au (>0.7 wt. %) would result in large nanoparticles and lead to decreased catalytic activity. Carbon nitride has N species similar with N-doped porous carbon which can stabilize Au atoms. Therefore, we employed the same method to deposit 0.2 wt. % Au on CNN membrane and successfully obtained well-dispersed single-site Au as shown in Figure 4b.

Our results show that the Au-CNN membrane is much more active in benzylamine oxidation than the CNN membrane (AQY~ 0), indicating that Au single sites play a critical role in the oxidation

reaction. It is accepted that the local electric field between single metal sites and coordinated N on the surface of carbon nitride drives the transfer of photogenerated electrons from carbon nitride to the single sites, which provide active sites for reduction reaction[7-9]. In our case, the photoexcited electrons in CNN could be captured by the surface Au atoms and activate oxygen to yield singlet oxygen ($^1O_2$, detected by EPR spectroscopy, Figure S17) and superoxide radical anion ($O_2^{\bullet-}$) as potentially important reactive species to enable benzylamine oxidation[10]. Therefore, we conclude that Au single sites could provide active sites for oxygen activation reactions.

Schemes

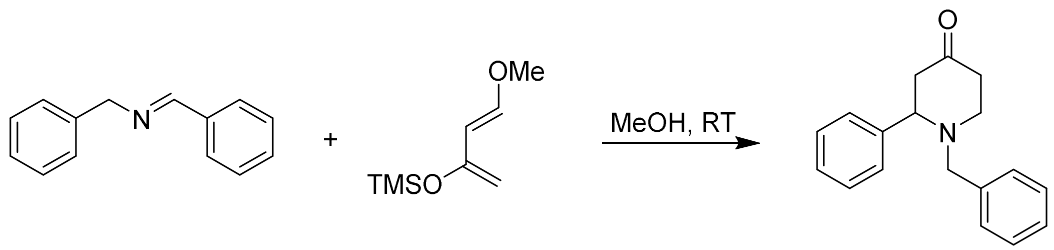

Scheme S1. Oxidative aza Diels-Alder reaction.

Figures

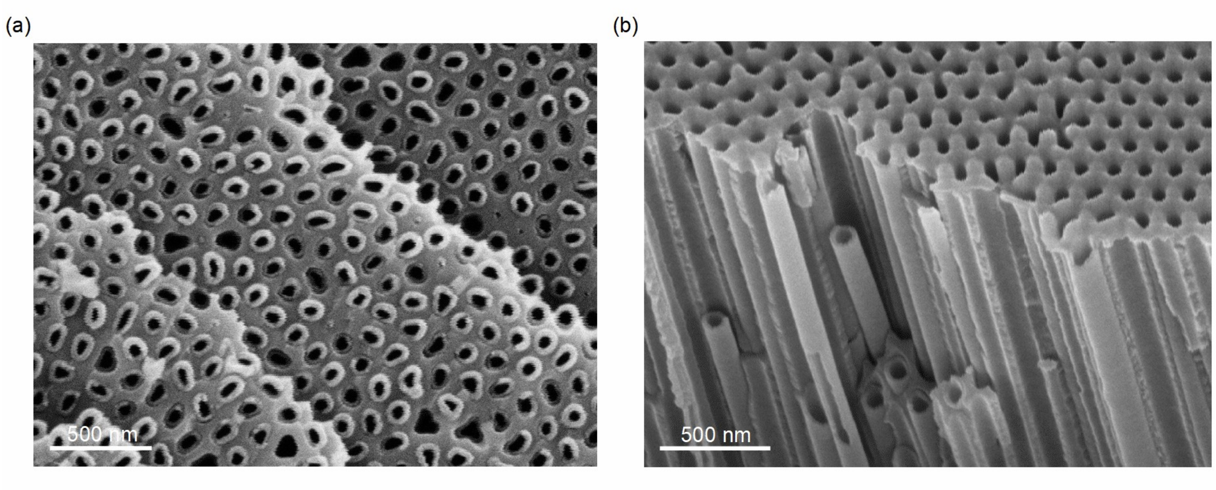

Figure S1. SEM images of CNN incorporated AAO membrane.

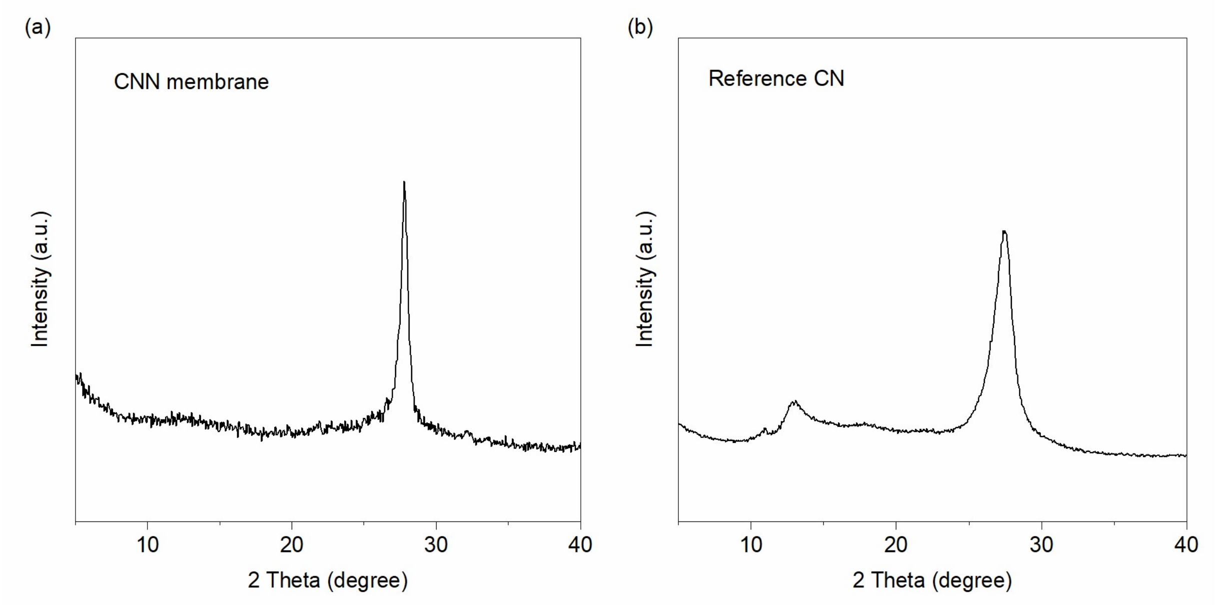

Figure S2. XRD patterns of the CNN membrane (a) and carbon nitride powder (b).

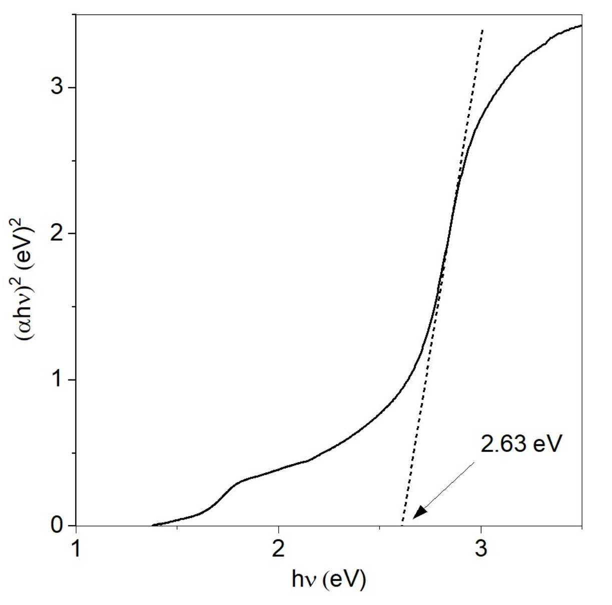

Figure S3. Tauc plot of the CNN membrane.

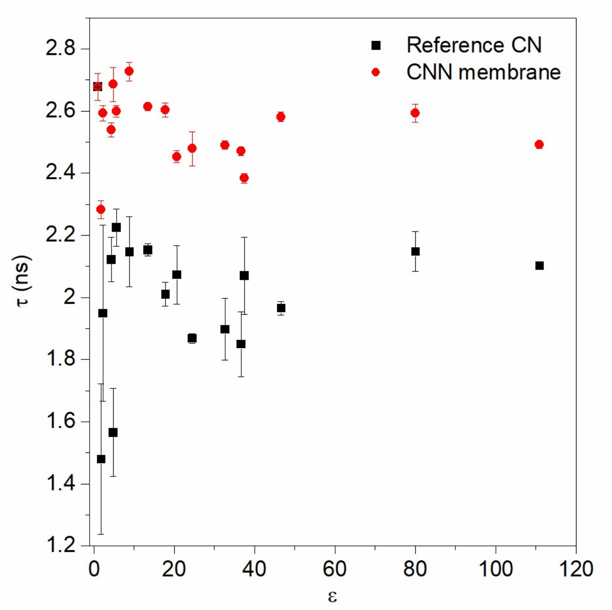

Figure S4. Dependence of carbon nitrides average fluorescence lifetime ($\lambda_{exc} = 375$ nm) on relative permittivity of the medium in which measurements have been conducted. Data points (from left to right) correspond to the following media: air (ε = 1), n-hexane (ε = 1.88), dioxane-1,4 (ε = 2.25), anisole (ε = 4.33), chloroform (ε = 4.81), chlorobenzene (ε = 5.62), dichloromethane (ε = 8.93), benzylalcohol (ε = 13.5), *iso*-propanol (ε = 17.9), acetone (ε = 20.7), ethanol (ε = 24.5), methanol (ε = 32.7), *N*,*N*-dimethylformamide (ε = 36.7), acetonitrile (ε = 37.5), dimethylsulfoxide (ε = 46.7), water (ε = 80.1), formamide (ε = 111).

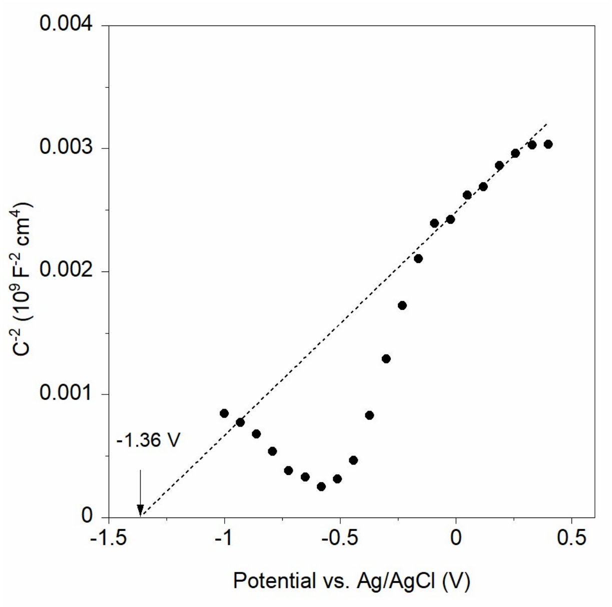

Figure S5. Mott-Schottky plot of the CNN membrane.

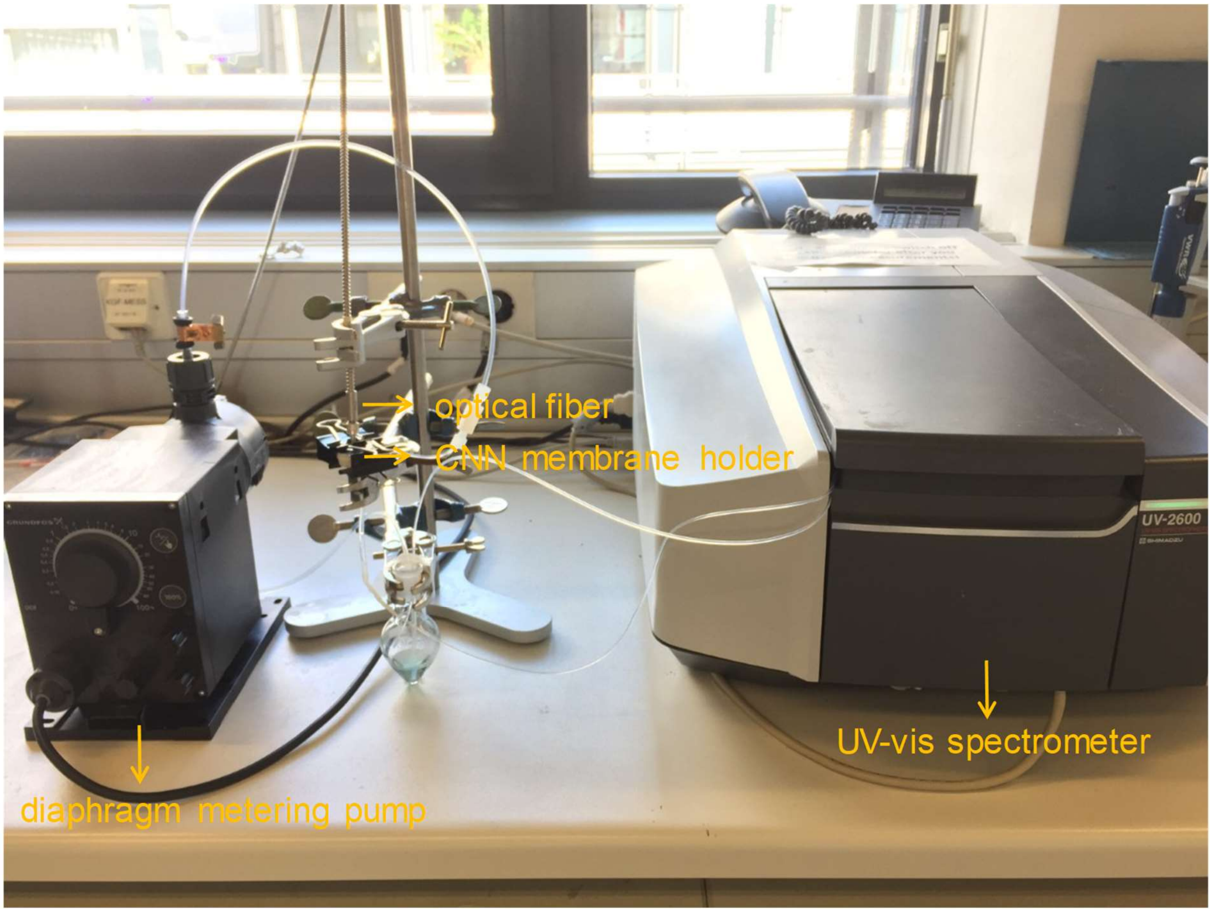

Figure S6. The photo of the set-up for MB degradation with the CNN membrane.

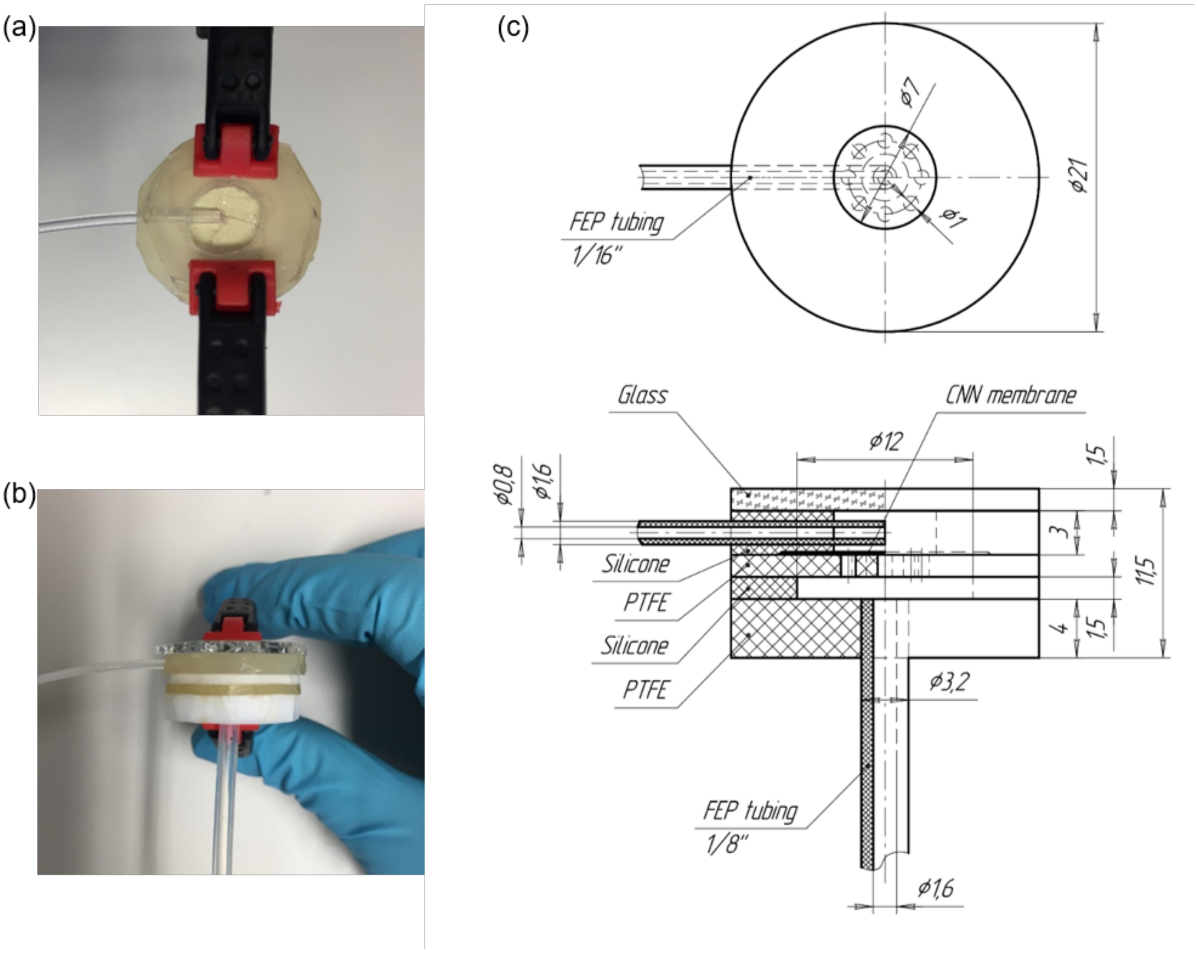

Figure S7. Top view (a) and side view (b) of the CNN membrane holder. (c) A schematic representation of the membrane holder. Scale 4:1.

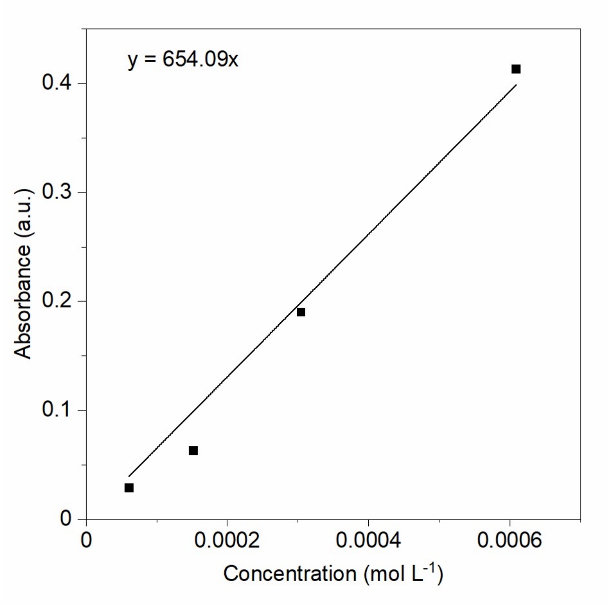

Figure S8. Calibration curve for the conversion of absorbance (a.u.) at 664 nm to concentration (mol L^{-1}).

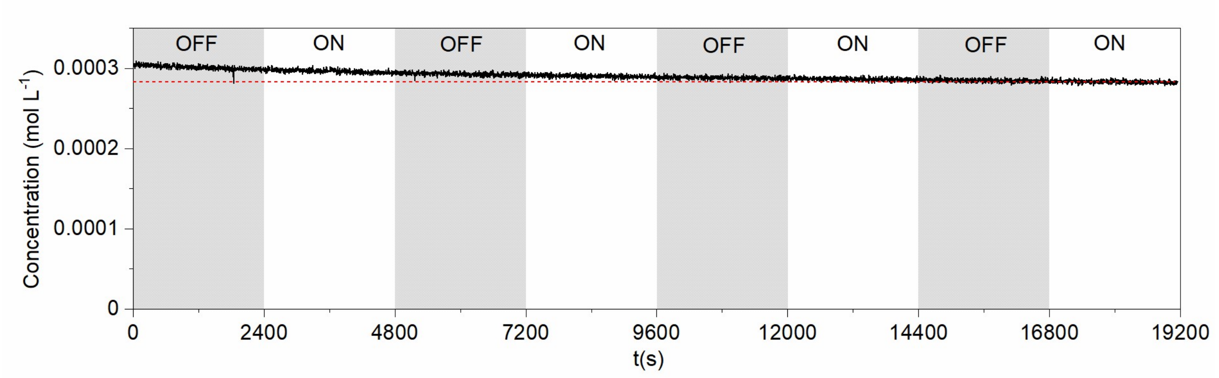

Figure S9. Decay curve of MB in the flow reactor without the CNN membrane.

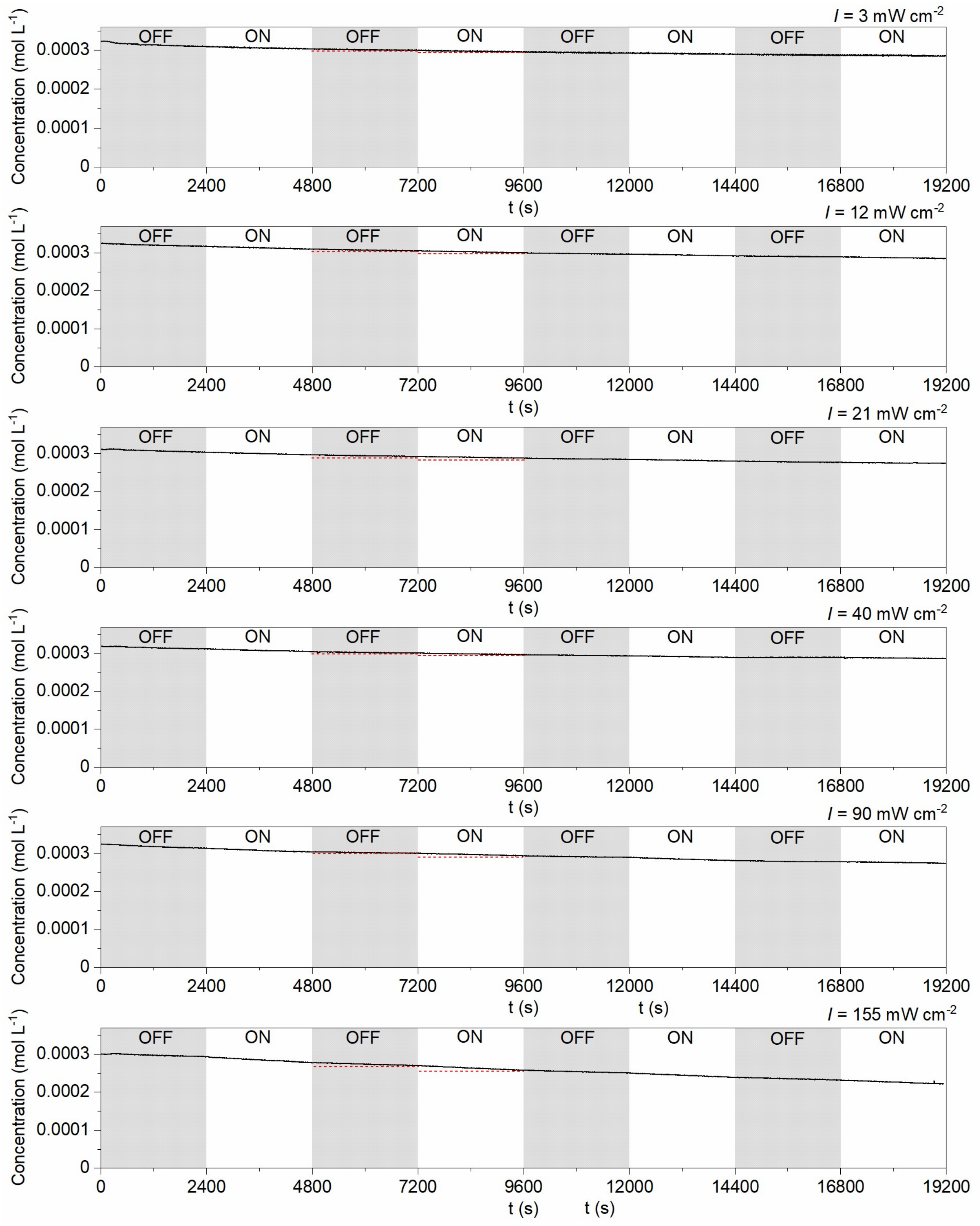

Figure S10. Decay curves of MB with different optical power (I).

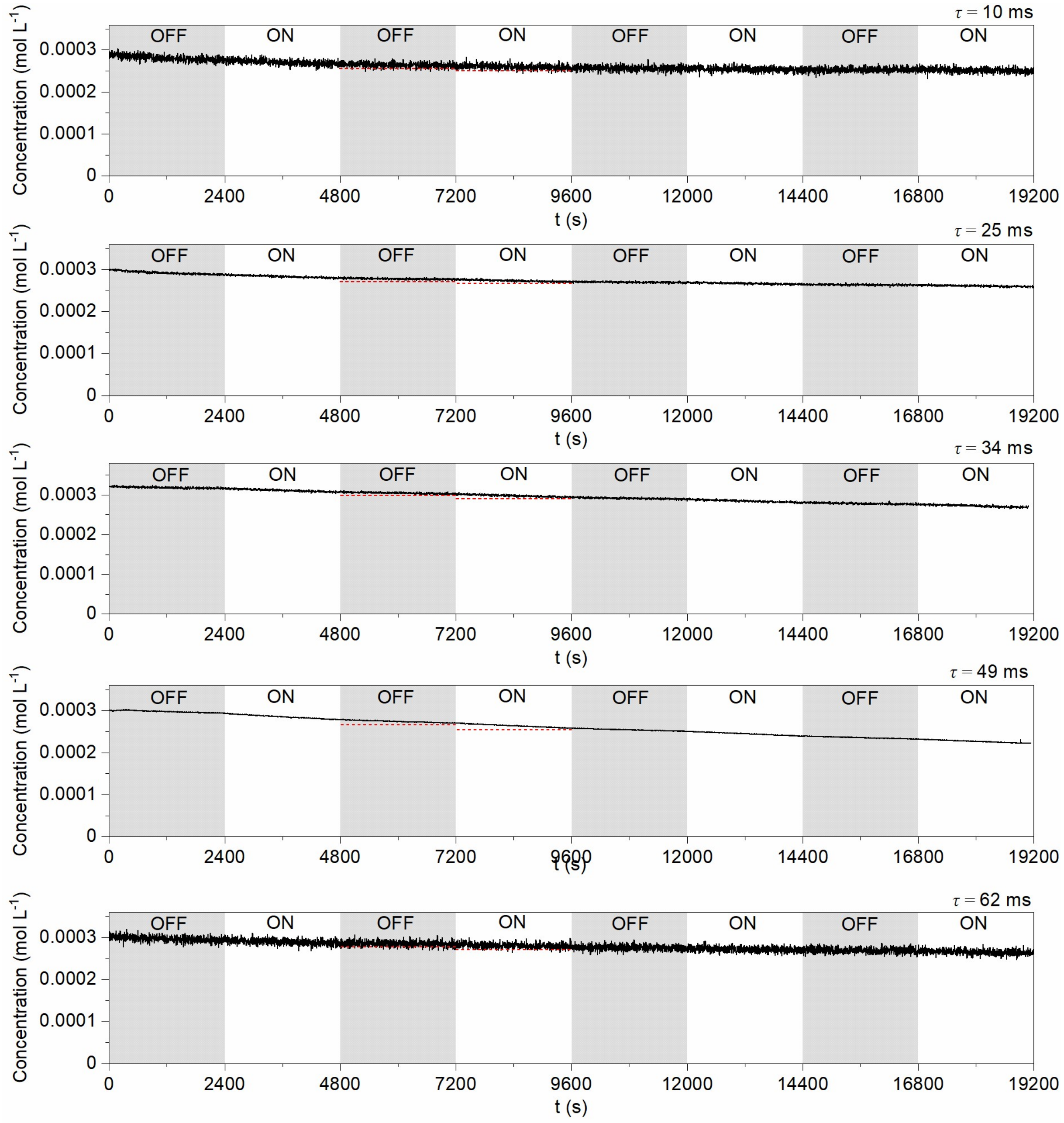

Figure S11. Decay curves of MB with different residence time (τ).

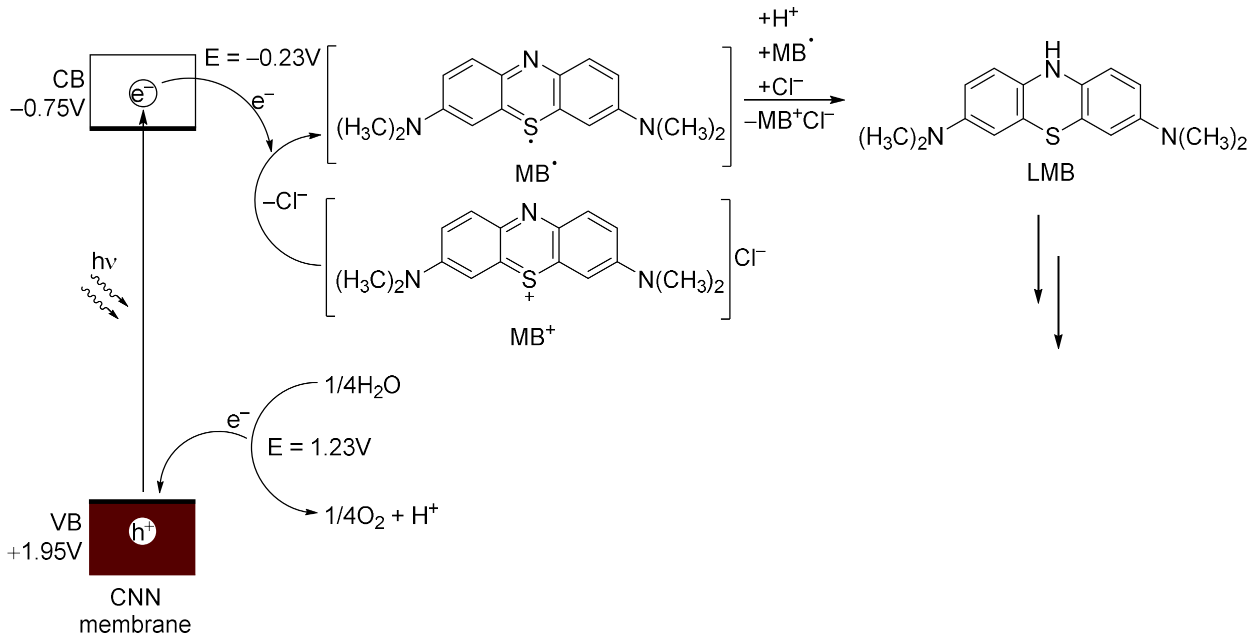

Figure S12. Proposed mechanism of methylene blue (MB^+Cl^-) degradation using the CNN membrane as a photoreactor.

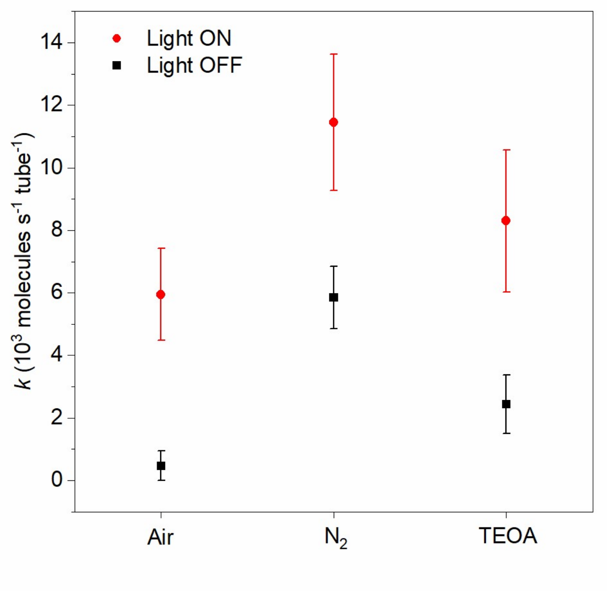

Figure S13. MB degradation rate in air, under anaerobic conditions and in the presence of triethanolamine (TEOA) as a sacrificial electron donor.

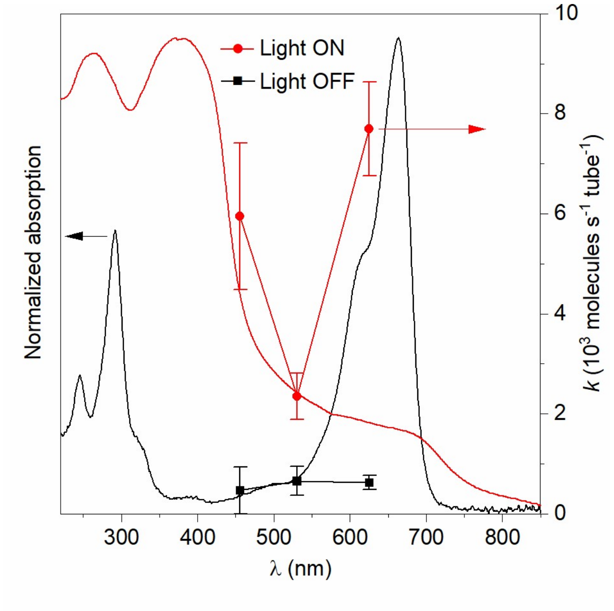

Figure S14. Correlation of light absorption by the CNN membrane and methylene blue with degradation rate.

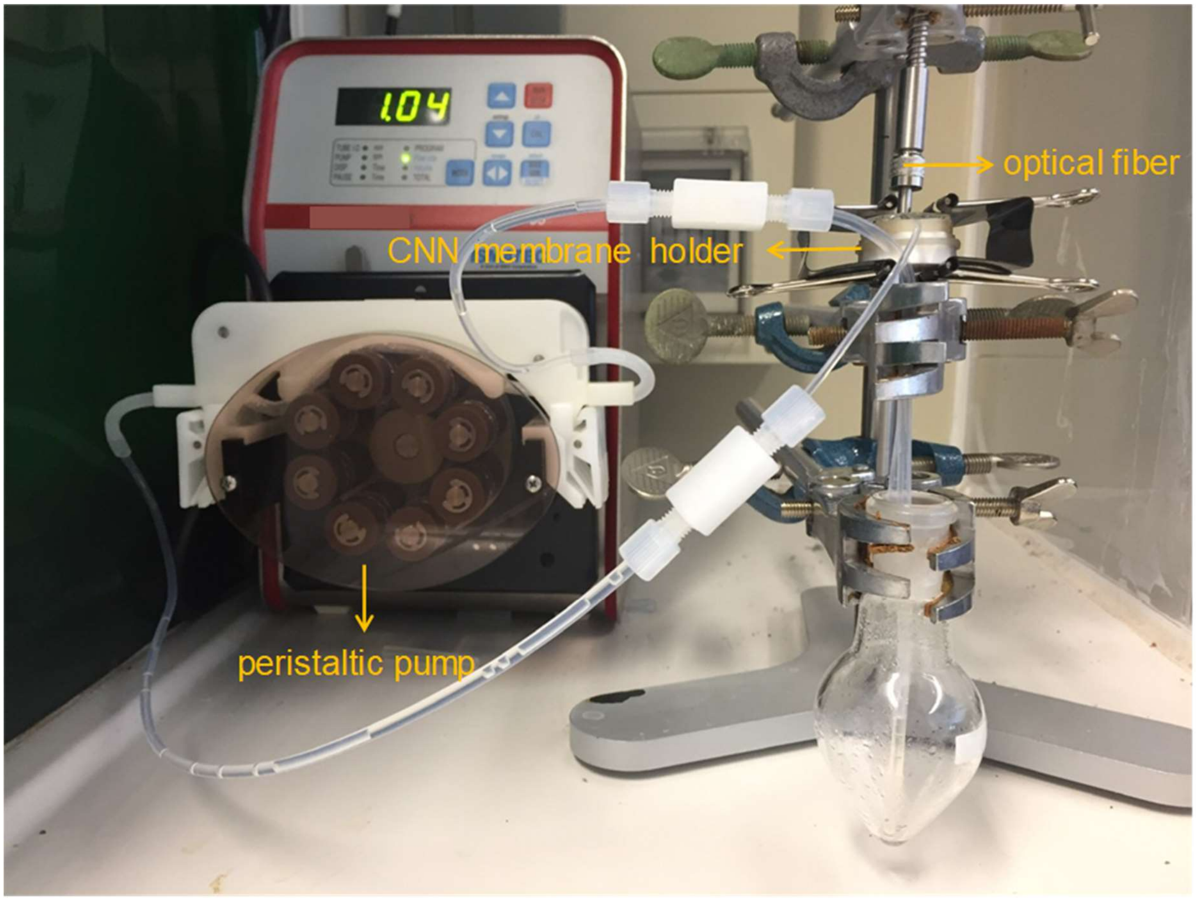

Figure S15. The photo of the set-up for amine oxidation with the Au-CNN membrane. Segmented flow was used to deliver O_2 to the Au-CNN membrane.

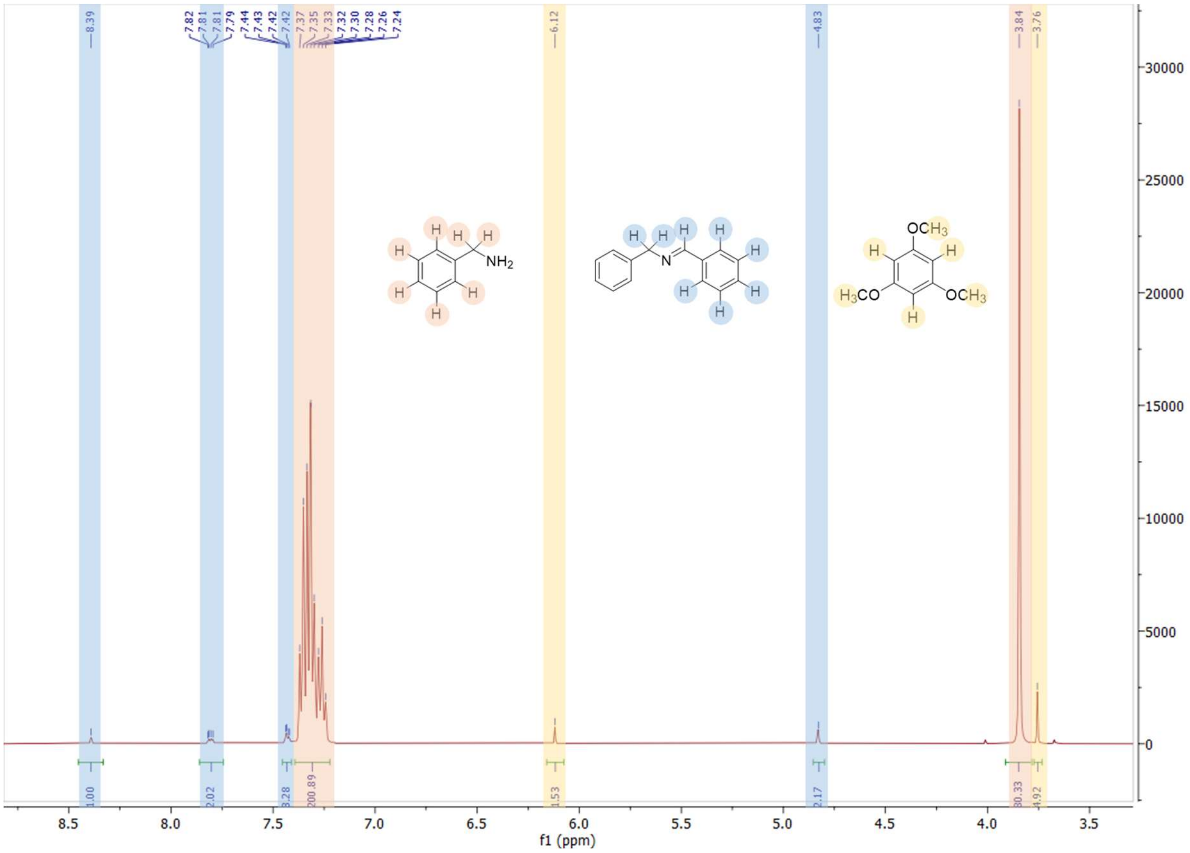

Figure S16. ^{1}H NMR spectrum of the reaction mixture of amine oxidation with the Au-CNN membrane under light irradiation. 1,3,5-trimethoxybenzene was used as internal standard.

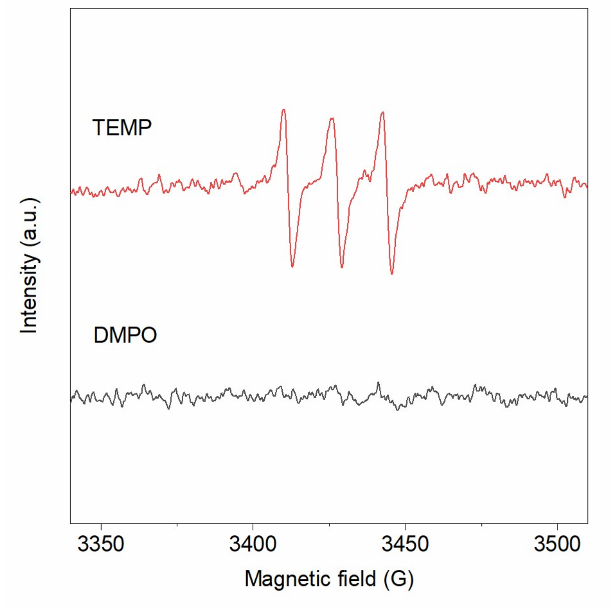

Figure S17. EPR spectra of ethanol containing TEMP or DMPO after flowing through the Au-CNN membrane under irradiation.

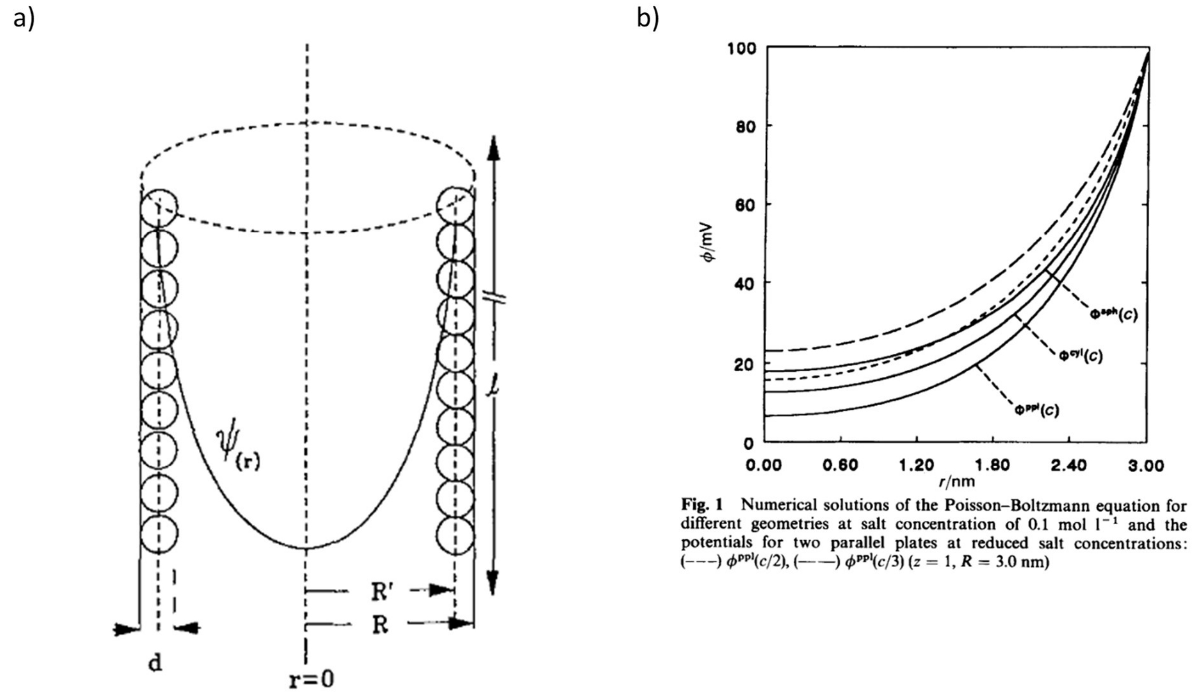

Figure S18. Profile of the electric field in the pore based on solving numerically Poisson-Boltzmann equation. (a) Reprinted from Journal of Colloid and Interface Science, 155, Sandra M. Sims, William I. Higuchi, V. Srinivasan, Kendall Peck, Ionic Partition Coefficients and Electroosmotic Flow in Cylindrical Pores: Comparison of the Predictions of the Poisson Boltzmann Equation with Experiment, 210-220, Copyright (1993), with permission from Elsevier. [11] (b) Reproduced from Ref. [12] with permission from The Royal Society of Chemistry.

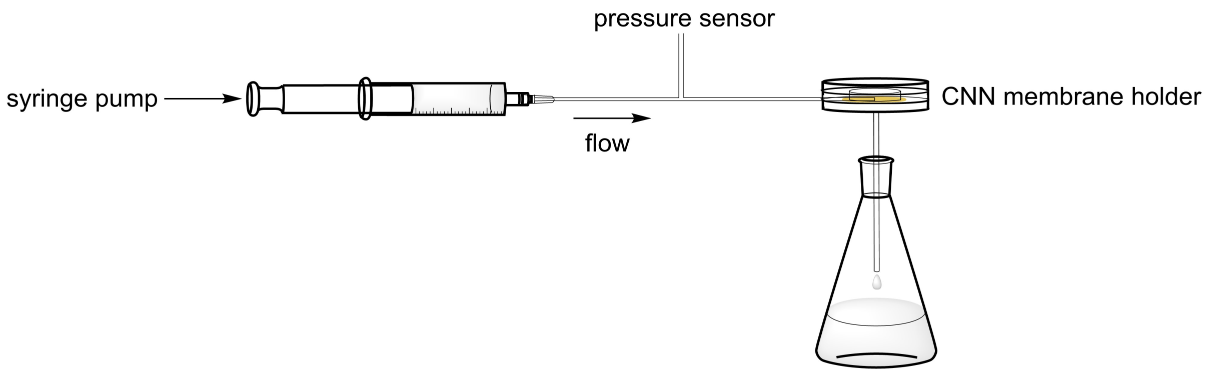

Figure S19. Schematic diagram of experimental setup for pressure measurement.

Tables

Table S1. Absorption coefficient (*A*) of the CNN membrane and the CN tablet

**Wavelength (nm)**	***A* of the CNN membrane (mW cm^{-3})[a]**	***A* of the CN tablet (mW cm^{-3})[a]**
350	82.494 ± 0.050	75.000 ± 0.000
400	144.474 ± 0.038	131.364 ± 0.000
410	143.975 ± 0.031	130.909 ± 0.000
450	157.966 ± 0.019	143.636 ± 0.000
500	99.426 ± 0.013	90.455 ± 0.000
525	121.225 ± 0.012	110.454 ± 0.000
550	136.318 ± 0.010	124.545 ± 0.000
600	112.585 ± 0.010	103.600 ± 0.009
650	85.750 ± 0.000	79.386 ± 0.007
700	59.370 ± 0.000	55.209 ± 0.006
750	43.374 ± 0.006	40.618 ± 0.005
800	25.962 ± 0.005	24.410 ± 0.004
850	12.118 ± 0.004	11.414 ± 0.004
900	43.754 ± 0.005	41.250 ± 0.004

[a] Mean ± std. dev. (n = 3)

Table S2. Reaction conditions in this work and some other reports

Ref.	MB concentration (μM)	MB solution volume (mL)	Photocatalyst	Catalyst mass (mg)	Light source
This work	3	4	g-C_3N_4	5	λ = 455 nm, 155 mW cm^{-2}
13	50	20	carbonaceous TiO_2	20	UV light
14	10	50	$MnTiO_3$	5	sunlight
15	125	30	PoPD-TiO_2	30	Xe lamp, 1000 W
16	31	100	Fe_2TiO_5	50	sunlight
17	31	200	cellulose/GO/TiO_2	5000	λ = 312 nm, 125 W
18	78	250	Au or Pt-TiO_2	250	UV-vis, 120 W m^{-2}
19	10	40	TiO_2	180	λ = 360 nm, 10 W m^{-2}

Table S3. TON, TOF and AQY of benzylamine oxidation with the Au-CNN membrane[a]

Entry	TON (10^{10} $tube^{-1}$)[b]	TOF (10^5 s^{-1} $tube^{-1}$)[b]	AQY (%)[b]
1	8.31 ± 1.61	9.63 ± 1.87	1.9 ± 0.7
2[c]	/	/	0.2 ± 0.1
3[d]	4.42 ± 0.26	5.12 ± 0.30	/
4[e]	/	/	1.0 ± 0.1

[a] Reaction conditions: 2 mL benzylamine, Au-CNN membrane photocatalyst, room temperature, λ = 465 nm. [b] Determined by ^{1}H NMR with 1,3,5-trimethoxybenzene as internal standard. Mean ± std. dev. (n = 3) [c] Without photocatalyst. [d] Without light irradiation. [e] With Au@CN instead of the Au-CNN membrane.

Table S4. Comparison of the CNN membrane performance in MB degradation in flow with some reported photocatalysts in batch.

Ref	Catalyst	S_{BET}	Pore volume, cm^3 [a]	Degradation rate	Degradation rate (molecules m^{-2} s^{-1})	MB solution volume, cm^3	Pore volume/ Reactor volume[b]
this work	CNN membrane	2.51×10^{-19} m^2 $tube^{-1}$	0.012	2308 molecules s^{-1} $tube^{-1}$	9.19×10^{21}	0.012	1
13	carbonaceous TiO_2	178.57 m^2 g^{-1}	0.00074	98.3% in 2h	2.30×10^{10}	20	$4\cdot10^{-5}$
15	PoPD-TiO_2	212.60 m^2 g^{-1}	0.019	18.0% in 1h[c]	1.77×10^{10}	30	$6\cdot10^{-4}$
16	Fe_2TiO_5	11.8 m^2 g^{-1}	0.0054	16.5% in 1h[d]	1.45×10^{11}	100	$5\cdot10^{-5}$
18	Au or Pt-TiO_2	53 m^2 g^{-1}	–	4.31×10^{-3} mg of methylene blue L^{-1} s^{-1}	1.53×10^{14}	250	–
19	TiO_2	–	–	68 μmol m^{-2} h^{-1}[e]	1.14×10^{13}	40	–

[a] Calculated as a product of specific pore volume (cm^3 g^{-1}) and mass of the photocatalyst (g) taken for degradation of methylene blue using data from the corresponding reference

[b] Calculated as a ratio between pore volume (cm^3) and volume of the methylene blue solution (cm^3)

[c] Calculated from first order rate constant 0.0033 min^{-1}

[d] Calculated from first order rate constant 0.003 min^{-1}

[e] Irradiation area instead of the S_{BET} area of catalyst

Table S5. Debye length for different electrolytes.

Entry	Electrolyte	Dielectric constant	Ionic strength, mol L^{-1}	l_B, nm	λ_D, nm
1	Pure water	80	10^{-7}	0.7	754
2	Aqueous methylene blue solution	80	$3.2 \cdot 10^{-7}$[a]	0.7	133
3	Benzylamine	4.6	–	12	$>10^5$[b]

[a] The following concentration of ions has been used to calculate the ionic strength of the electrolyte: $[H^+] = [HO^-] = 10^{-7}$ mol L^{-1}; [MB cation] = $[Cl^-] = 3.125 \cdot 10^{-7}$ mol L^{-1}

[b] Due to low acidity of benzylamine, λ_D has been arbitrary assigned to $>10^5$ nm, to emphasize that it is significantly longer than λ_D in aqueous environment

Table S6. Measured zeta-potential of the CNN membrane and reference carbon nitride in different electrolytes. Calculated surface potential and electric field gradient.

Entry	Electrolyte	Material	ζ, mV	ϕ_0, mV	E, V m^{-1}[b]
1	Pure water	Reference carbon nitride	–38.9±0.7	–105.4	$7 \cdot 10^5$
2	Aqueous methylene blue solution[a]	Reference carbon nitride	–38.3±0.6	–103.8	$7 \cdot 10^5$
3	Pure water	CNN membrane	–22.4±1.3	–60.7	$8 \cdot 10^4$
4	Aqueous methylene blue solution[a]	CNN membrane	–14.8±0.5	–40.1	$5 \cdot 10^4$

[a] MB concentration in water $3.125 \cdot 10^{-6}$ M

[b] At distance 20 nm from the surface.

Supplementary references

(1) Xiao, K.; Chen, L.; Chen, R.; Heil, T.; Lemus, S. D. C.; Fan, F.; Wen, L.; Jiang, L.; Antonietti, M., Artificial Light-Driven Ion Pump for Photoelectric Energy Conversion. *Nat. Commun.* **2019,** *10*, 74.

(2) Qu, C.; Myllymaa, S.; Prittinen, J.; Koistinen, A. P.; Lappalainen, R.; Lammi, M. J., Osteoblast Behavior on Various Ultra Short Pulsed Laser Deposited Surface Coatings. *Mater. Sci. Eng. C* **2013,** *33*, 1676-1682.

(3) Kokot, G.; Bespalova, M. I.; Krishnan, M., Measured Electrical Charge of SiO_2 in Polar and Nonpolar Media. *J. Chem. Phys.* **2016,** *145*, 194701.

(4) Mills, A.; Wang, J., Photobleaching of Methylene Blue Sensitised by TiO_2: An Ambiguous System? *J.Photochem. Photobiol. A* **1999,** *127*, 123-134.

(5) Nassar, S. J. M.; Wills, C.; Harriman, A., Inhibition of the Photobleaching of Methylene Blue by Association with Urea. *ChemPhotoChem* **2019,** *3*, 1042-1049.

(6) Qin, Q.; Heil, T.; Antonietti, M.; Oschatz, M., Single-Site Gold Catalysts on Hierarchical N-Doped Porous Noble Carbon for Enhanced Electrochemical Reduction of Nitrogen. *Small Methods* **2018,** *2*, 1800202.

(7) Yang, Y.; Li, F.; Chen, J.; Fan, J.; Xiang, Q., Single Au Atoms Anchored on Amino-Group-Enriched Graphitic Carbon Nitride for Photocatalytic CO_2 Reduction. *ChemSusChem* **2020,** *13*, 1979-1985.

(8) Cao, S.; Li, H.; Tong, T.; Chen, H.-C.; Yu, A.; Yu, J.; Chen, H. M., Single-Atom Engineering of Directional Charge Transfer Channels and Active Sites for Photocatalytic Hydrogen Evolution. *Advanced Functional Materials* **2018,** *28*, 1802169.

(9) Gao, G.; Jiao, Y.; Waclawik, E. R.; Du, A., Single Atom (Pd/Pt) Supported on Graphitic Carbon Nitride as an Efficient Photocatalyst for Visible-Light Reduction of Carbon Dioxide. *J Am Chem Soc* **2016,** *138*, 6292-6297.

(10) Kumar, R.; Gleissner, E. H.; Tiu, E. G.; Yamakoshi, Y., C_{70} as a Photocatalyst for Oxidation of Secondary Benzylamines to Imines. *Org. Lett.* **2016,** *18*, 184-187.

(11) Sims, S. M.; Higuchi, W. I.; Srinivasan, V.; Peck, K., Ionic Partition Coefficients and Electroosmotic Flow in Cylindrical Pores: Comparison of the Predictions of the Poisson-Boltzmann Equation with Experiment. *J. Colloid Interface Sci.* **1993,** *155*, 210-220.

(12) Yang, Y.; Walz, J.; Pintauro, P., Curvature Effects on Electric Double-Layer Forces. Part 1.—Comparisions with Parallel Geometry. *J. Chem. Soc. Faraday Trans.* **1995,** *91*, 2827-2836.

(13) Ramli, Z. A.; Asim, N.; Isahak, W. N.; Emdadi, Z.; Ahmad-Ludin, N.; Yarmo, M. A.; Sopian, K., Photocatalytic Degradation of Methylene Blue under UV Light Irradiation on Prepared Carbonaceous TiO_2. *Sci. World J.* **2014,** *2014*, 415136.

(14) Alkaykh, S.; Mbarek, A.; Ali-Shattle, E. E., Photocatalytic Degradation of Methylene Blue Dye in Aqueous Solution by $MnTiO_3$ Nanoparticles under Sunlight Irradiation. *Heliyon* **2020,** *6*, e03663.

(15) Yang, C.; Dong, W.; Cui, G.; Zhao, Y.; Shi, X.; Xia, X.; Tang, B.; Wang, W., Highly-Efficient Photocatalytic Degradation of Methylene Blue by PoPD-Modified TiO_2 Nanocomposites due to Photosensitization-Synergetic Effect of TiO_2 with PoPD. *Sci. Rep.* **2017,** *7*, 3973.

(16) Vasiljevic, Z. Z.; Dojcinovic, M. P.; Vujancevic, J. D.; Jankovic-Castvan, I.; Ognjanovic, M.; Tadic, N. B.; Stojadinovic, S.; Brankovic, G. O.; Nikolic, M. V., Photocatalytic Degradation of Methylene Blue under Natural Sunlight Using Iron Titanate Nanoparticles Prepared by a Modified Sol-Gel Method. *Roy. Soc. Open Sci.* **2020,** *7*, 200708.

(17) Chen, Y.; Xiang, Z.; Wang, D.; Kang, J.; Qi, H., Effective Photocatalytic Degradation and Physical Adsorption of Methylene Blue Using Cellulose/GO/TiO_2 Hydrogels. *RSC Adv.* **2020,** *10*, 23936-23943.
(18) Mesa, J. J. M. R., J. R. G.; Macías, Á. C. C.; Sarmiento, H. A. R.; Lobo, J. A. C.; López, M. C. H.; Santos, J. A. N., Methylene Blue Degradation over M-TiO_2 photocatalysts (M= Au or Pt). *Ciencia en Desarrollo* **2017,** *8*, 109-117.
(19) Tschirch, J.; Dillert, R.; Bahnemann, D.; Proft, B.; Biedermann, A.; Goer, B., Photodegradation of Methylene Blue in Water, a Standard Method to Determine the Activity of Photocatalytic Coatings? *Res. Chem. Intermed.* **2008,** *34*, 381-392.
